# Supplementary material for: Effectiveness of an Innovative Sensory Approach to Improve Children’s Nutritional Choices
Source: Int J Environ Res Public Health. 2021 Jun 15;18(12):6462. doi: 10.3390/ijerph18126462 (PMC8296279; doi:10.3390/ijerph18126462)
Supplement: Supplementary file 1 [file ijerph-18-06462-s001.zip › ijerph-1210048-supplementary/Supplementary materials-S1.pdf]

SUPPLEMENTARY MATERIALS-S1

SECTION S1.1 Methods of carrying out the theoretical and practical lessons.

S1.1.1 Activity sequence in the classroom

1) Produce a billboard to hang in the classroom for scoring. In table 1, data assigned to each activity are indicated.

2) Each class was divided into two teams. Then, there was the assignment of team leaders drawing the two foremen to refer to the answers during the games

3) Having the nursery rhyme repeat to each team and score

4) Start of the laboratories and score the each team

5) Subsequently, teachers continued the classroom activities with three additional laboratories for three months following the study:

- Art image with fruit
- Art the image with the vegetable
- Sciences, in which children have seen how to make ricotta and yogurt from milk

Moreover, in June 2017, the world of the Govut was brought to the stage with a theatrical performance at the end of the year, in which children of all sample classes took part.

Doggerel	6 points
Olfattando	15 points
Investigator hand	15 points
Saporando	9 points
Apple theorem	15 points
Publicity	10 points
RefectoryDoggerel	30 points

Table S1. List of different laboratories and the score assigned for each activity.

S1.1.2 Main laboratory activities

- Doggerel: A very important moment was to teach the students a nursery rhyme about the right behavior at the table, which was repeated in class every day. 6 points were awarded to be placed immediately on the board.
- Olfattando: Children had to recognize five object's smells between spices and foods and, then, match the appropriate adjective of the *vocacibario* among the terms of OLFAT. Five plastic cups were used to cover with aluminum foil and perforate on the surface, so as not to show the contents. For each correct answer 1 point was awarded and for each correct adjective 2 points were awarded, for a total of 15 points.

  1.pepper, eg. adjective pungent

  2.basil, eg. adjective aromatic

  3.garlic, eg. adjective intense

  4.anchovyes, eg. adjective unpalatable

  5.cinnamon, eg. adjective spicy.
- Investigator hand: children had to guess the box's contents by touching five objects and, then, match the appropriate adjective of the *vocacibario* among the terms of TATTONE. Five dark boxes were used to not show the contents, in which children put their hand and guessed the contents, even blindfolded. For each correct answer 1 point was awarded and for each correct adjective 2 points were awarded, for a total of 15 points.

  1. dried prunes, eg. adjective sticky

  2. celery, eg. adjective smooth

  3. tangerine, eg. adjective wrinkled

4. carrot, eg. adjective scratchy

5. fennel, eg. adjective filamentous.

- Saporando: children were blindfolded and tasted 3 foods. The task was to recognize the food and attribute the appropriate adjective of the *vocacibario* among the terms of GUSTINO. For each correct answer 1 point was awarded and for each correct adjective, 2 points were awarded, for a total of 9 points. In this game the "tasting cards" of the Edueat®Method were used.

  1. fennel, eg. adjective sweet, fresh

  2. radish, eg. adjective spicy

  3. walnut, eg. adjective hard.

- Apple theorem: five types of apples were used. The characteristics of each apple have been illustrated. Afterwards, children had to recognize them tasting the different types of apples. Apples have been cut and placed in food bags. For each correct answer 1 point was awarded and for each correct adjective 2 points were awarded, for a total of 15 points

  1. red apple
  2. smith apple
  3. annurca apple
  4. golden apple
  5. pink lady apple.

- Vegetables championship: the game was performed during lunch in the school refractory, organizing it with the operators involved. The championship has been divided into three days.

  The first two days children awarded a winner among the vegetables in the race, proclaiming the semi-finalists. The voting took place by distributing to the children cards

with the reference vegetable. Each child has therefore voted putting in a box the most appreciated vegetables.

1. green beans vs cauliflower
2. spinach vs eggplant
3. the final: win the best.

S1.1.3 Example of the nursery rhyme

"Poesia & Pulizia"

Apri bene il rubinetto
senza bagnare il tuo giacchetto
Insapona le manine
che pulite son più carine
Sfrega i palmi tra di loro
brilleranno come l'oro
Passa bene tra le dita
la tua mano sarà più pulita
Strofinando dentro e fuori
si cancellano anche i colori
Intorno ai pollici gira bene
che lo sai che il Govut ci tiene
Sciacqua sciacqua forte e in fretta
c'è la cena che ti aspetta!"

SECTION S1.2 Questionnaire

S1.2.1 Italian version

Qui sotto troverai delle domande riguardo la tua alimentazione e le tue preferenze per i cibi. Ti assicuriamo che le risposte che darai non saranno giudicate giuste o sbagliate, né saranno fatte vedere ai tuoi genitori. Quindi, ti preghiamo di leggere tutto e di rispondere con calma e sincerità. Grazie mille!

La tua data di nascita è ___/___/____

Sei maschio o femmina? ☐ M ☐ F

Questionario A)

**Rifletti e rispondi alle affermazioni seguenti:**	**Vero**	**Falso**
A1. Mangio soprattutto cibi ricchi e appetitosi	☐	☐
A2. E’ molto difficile farmi variare l’alimentazione	☐	☐
A3. Mi piace fare tanti piccoli spuntini nell’arco della giornata	☐	☐
A4. Mi piace un’alimentazione molto varia	☐	☐
A5. Farmi mangiare è sempre un’impresa molto difficile	☐	☐
A6. Bado più alla quantità che alla qualità del cibo	☐	☐
A7. Amo mangiare sempre le stesse cose	☐	☐
A8. Mangio soprattutto fuori pasto	☐	☐
A9. Mangio volentieri tutti ciò che mi danno	☐	☐
A10. E’ necessario usare molta fantasia per stimolarmi a mangiare	☐	☐

Questionario B)

TI PIACE	Molto	Così così	Per niente	TI PIACE	Molto	Così così	Per niente
B01. Legumi(piselli, fagioli…)				B17. Banane			
B02. Broccoli				B18. Kiwi			
B03. Carciofi				B19. Frutti di bosco			
B04. Carote				B20. Frutta esotica			
B05. Cavolfiore				B21. Frutta secca con guscio			
B06. Pomodoro				B22. Pera/Mela			
B07. Fagiolini				B23. Melone			
B08. Finocchi				B24. Uva			
B09. Funghi				B25. Albicocche Pesche			
B10. Insalata				B26. Bastoncini			
B11. Peperone				B27. Gamberi/ Gamberetti			
B12. Patata				B28. Sogliola/Platessa			
B13. Sedano				B29. Vongole, Cozze			
B14. Spinaci				B30. Pesce: trancio/filetto			
B15. Zucchine				B31. Polpo Seppia			

B16. Agrumi				B32. Tonno in scatola			

Questionario C)

**Per favore scegli una risposta per ogni cibo o bevanda, mettendo una crocetta sul quadratino scelto**	**FA MOLTO BENE ALLA SALUTE**	**FA BENE ALLA SALUTE**	**NON FA BENE**
C01. Acqua	☐	☐	☐
C02. Bibite zuccherate (aranciata, cola, tè)	☐	☐	☐
C03. Biscotti	☐	☐	☐
C04. Burro	☐	☐	☐
C05. Dolciumi (caramelle, gomme)	☐	☐	☐
C06. Carne	☐	☐	☐
C07. Cereali per la colazione	☐	☐	☐
C08. Cioccolata - Crema di cioccolata	☐	☐	☐
C09. Formaggio	☐	☐	☐
C10. Frutta	☐	☐	☐
C11. Gelato	☐	☐	☐
C12. Hamburger (fast food)	☐	☐	☐
C13. Latte / Yogurt	☐	☐	☐
C14. Legumi (ceci, piselli, lenticchie..)	☐	☐	☐
C15. Merendine	☐	☐	☐
C16. Minestrone	☐	☐	☐
C17. Olio d’oliva	☐	☐	☐
C18. Pane	☐	☐	☐
C19. Pasta, Riso	☐	☐	☐

C20. Patate	☐	☐	☐
C21. Patatine confezionate	☐	☐	☐
C22. Pesce	☐	☐	☐
C23. Pizza	☐	☐	☐
C24. Prosciutto cotto	☐	☐	☐
C25. Prosciutto crudo	☐	☐	☐
C26. Salame/Mortadella	☐	☐	☐
C27. Succo di frutta	☐	☐	☐
C28. Torte	☐	☐	☐
C29. Uova	☐	☐	☐
C30. Verdura cruda	☐	☐	☐
C31. Verdura cotta	☐	☐	☐
C32. Vino	☐	☐	☐

S1.2.2 English version

Below you will find questions about your diet and food preferences. We assure you that the answers you give will not be judged right or wrong, nor will they be shown to your parents. So, please read everything and respond calmly and sincerely. Thanks so much!

Your date of birth is ___/___/____

Are you male or female? ☐ M ☐ F

Questionnaire A)

Ponder and respond to the following statements:	True	False
A1. I mostly eat rich and appetizing foods	☐	☐
A2. It is very difficult to make me vary my diet	☐	☐
A3. I like to have many small snacks throughout the day	☐	☐
A4. I like a very varied diet	☐	☐
A5. Getting me to eat is always a very difficult undertaking	☐	☐
A6. I pay more attention to the quantity than to the quality of the food	☐	☐
A7. I love to always eat the same things	☐	☐
A8. I mostly eat between meals	☐	☐
A9. I gladly eat everything they give me	☐	☐
A10. It is necessary to use a lot of imagination to stimulate me to eat	☐	☐

Questionnaire B)

YOU LIKE	very much	So and so	for nothing	YOU LIKE	very much	So and so	for nothing
B01. Legumes (peas, beans ...)				B17. Bananas			

B02. Broccoli				B18. Kiwi			
B03. Artichokes				B19. Berries			
B04. Carrots				B20. Esotic fruit			
B05. Cauliflower				B21. Dried fruit in shell			
B06. Tomato				B22. Pear / Apple			
B07. Green beans				B23. Melon			
B08. Fennel				B24. Grapes			
B09. Mushrooms				B25. Apricots / Peaches			
B10. Salad				B26. Sticks			
B11. Sweet pepper				B27. Shrimp / Shrimp			
B12. Potato				B28. Sole / Plaice			
B13. Celery				B29. Clams, / Mussels			
B14. Spinach				B30. Fish: steak / fillet			
B15. Zucchini				B31. Octopus / Sepia			
B16. Citrus fruits				B32. Canned tuna			

Questionnaire C)

**Please choose an answer for each food or drink, placing a cross on the chosen square**	**IT IS VERY GOOD**	**IT'S HEALTHY**	**NOT GOOD FOR HEALTH**

	**FOR HEALTH**		
C01. Water	☐	☐	☐
C02. Sugary drinks (orange soda, cola, tea)	☐	☐	☐
C03. Cookies	☐	☐	☐
C04. Butter	☐	☐	☐
C05. Sweets (candy, gum)	☐	☐	☐
C06. Meat	☐	☐	☐
C07. Breakfast cereals	☐	☐	☐
C08. Chocolate - Chocolate cream	☐	☐	☐
C09. Cheese	☐	☐	☐
C10. Fruit	☐	☐	☐
C11. Ice cream	☐	☐	☐
C12. Hamburger (fast food)	☐	☐	☐
C13. Milk / Yogurt	☐	☐	☐
C14. Legumes (chickpeas, peas, lentils ..)	☐	☐	☐
C15. Snacks	☐	☐	☐
C16. Vegetable soap	☐	☐	☐
C17. Olive oil	☐	☐	☐
C18. Bread	☐	☐	☐
C19. Pasta, Rice	☐	☐	☐
C20. Potatoes	☐	☐	☐
C21. Potato chips	☐	☐	☐
C22. Fish	☐	☐	☐
C23. Pizza	☐	☐	☐

C24. Cooked ham	☐	☐	☐
C25. Raw ham	☐	☐	☐
C26. Salami / Mortadella	☐	☐	☐
C27. Fruit juice	☐	☐	☐
C28. Cakes	☐	☐	☐
C29. Eggs	☐	☐	☐
C30. Raw vegetables	☐	☐	☐
C31. Cooked vegetables	☐	☐	☐
C32. Wine	☐	☐	☐
