# Supplementary material for: Effectiveness of an Innovative Sensory Approach to Improve Children’s Nutritional Choices
Source: Int J Environ Res Public Health. 2021 Jun 15;18(12):6462. doi: 10.3390/ijerph18126462 (PMC8296279; doi:10.3390/ijerph18126462)
Supplement: Supplementary file 1 [file ijerph-18-06462-s001.zip › ijerph-1210048-supplementary/Supplementary materials-S2.pdf]

CONFERENZA
TERRITORIALE
SOCIALE E SANITARIA

PIANO PER LA SALUTE

SERVIZIO SANITARIO REGIONALE
EMILIA-ROMAGNA
Azienda Unità Sanitaria Locale di Cesena

Sorveglianza nutrizionale nelle scuole elementari dell'Azienda Sanitaria di Cesena

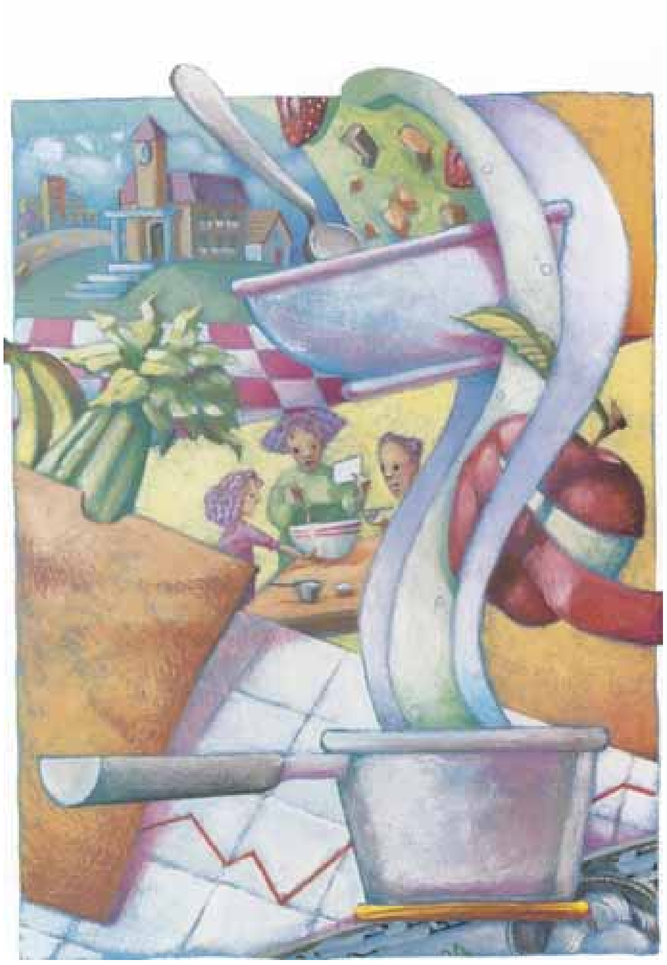

A cura di: Roberta Cecchetti, Nicoletta Bertozzi, Francesca Castoldi, Davide Lazzarini, Mauro Palazzi, Vannia Ricci, Francesca Righi, Ruggero Ruggeri, Patrizia Vitali

(Dipartimento di Sanità Pubblica Ausl di Cesena)

Elena Prati, Chiara Reali

(Alma Mater Studiorum - Università di Bologna - Scuola di Specializzazione in Igiene e Medicina Preventiva)

Augusta Albertini, Francesca Celenza, Valeria Contarini, Claudia Cortesi, Elena Dalle Donne, Simonetta De Giorgi, Emanuela Di Martino, Alessandra Fabbri, Marta Mattioli, Giuseppe Melandri, Annamaria Rauti, Angelo Rossi, Chiara Rizzoli, Cristina Saletti, Ivana Stefanelli, Alberto Tripodi, Bruna Veronesi

(Gruppo Nutrizione Regione Emilia Romagna)

Giulia Cavrini, Manuela Nicolucci

(Alma Mater Studiorum - Università di Bologna – Dipartimento di Scienze Statistiche)

Hanno collaborato alla realizzazione dello studio:

Antonella Brunelli
Massimo Farneti
(U.O. Materno Infantile Ausl di Cesena)
Gabriella Dallara
(Dipartimento di Sanità Pubblica Ausl di Cesena)

Le scuole elementari del territorio dell'Azienda Sanitaria di Cesena

Per i dati regionali tutte le Aziende Sanitarie della Regione Emilia Romagna che hanno aderito allo studio

Si rivolge un sincero ringraziamento ai bambini e ai genitori che hanno partecipato all'indagine

Copia della presente pubblicazione può essere richiesta a:
Roberta Cecchetti
Tel.0541-946593
e-mail: rcecchetti@ausl-cesena.emr.it

Febbraio 2007

Indice

Presentazione 7

Introduzione 8

Materiali e metodi 9

Sintesi 12

Descrizione del campione 17

Sovrappeso e obesità 20

Attività fisica 23

La giornata alimentare tipo 24

Le frequenze di consumo degli alimenti 31

Le abitudini alimentari 34

I bambini e l'alimentazione 39

Igiene orale 44

Conclusioni 46

Bibliografia……………………………………………………………………………… 47

Allegato 1 Questionario somministrato ai genitori dei bambini di 6 anni

Allegato 2 Questionario somministrato ai genitori dei bambini di 9 anni

Allegato 3 Questionario somministrato ai bambini di 9 anni

Presentazione

In tutti i paesi del mondo, industrializzati e anche in via di sviluppo, negli ultimi decenni, si è assistito ad un vero e proprio incremento delle malattie cronico-degenerative non trasmissibili, diventate le maggiori responsabili della mortalità generale.

In particolare per l'obesità l'Organizzazione Mondiale della Sanità (OMS) parla di epidemia in quanto la sua prevalenza è aumentata di tre volte nelle ultime due decadi; in Europa metà di tutti gli adulti e un bambino su cinque sono sovrappeso.

La tendenza è particolarmente allarmante nei bambini e negli adolescenti, poiché in questo modo l'epidemia si sposta nell'età adulta e genera un progressivo peggioramento della salute per le generazioni future. Sovrappeso ed obesità affliggono principalmente le persone di classe socioeconomica bassa e questo contribuisce ad aumentare le diseguaglianze nello stato di salute.

L'epidemia si è ampliata negli ultimi decenni a causa dei cambiamenti dell'ambiente culturale, sociale, economico e fisico. Uno squilibrio energetico nella popolazione è stato innescato da una drammatica riduzione dell'attività fisica e dai mutamenti negli schemi dietetici, incluso l'incremento nel consumo di cibi e bevande ad alta densità energetica e poveri di nutrienti in combinazione con un basso consumo di frutta e vegetali.

Secondo l'OMS è possibile però invertire l'andamento e tenere l'epidemia sotto controllo attraverso efficaci strategie di prevenzione.

Lo sviluppo di una strategia che porti ad un aumento del livello di attività fisica così come all'adozione di corrette abitudini alimentari all'interno della popolazione, mediante l'adozione di interventi di dimostrata efficacia, è un obiettivo di sanità pubblica che può essere raggiunto solo attraverso politiche sanitarie (interagenti con politiche di altri settori), condivisioni di obiettivi e individuazione delle diverse responsabilità.

Per creare una società dove gli stili di vita salutari per dieta e attività fisica, siano la norma è essenziale costruire collaborazione a tutti i livelli (governi, società civile, il settore privato, le reti di professionisti, i media e le organizzazioni internazionali); non è accettabile lasciare che gli individui siano i soli responsabili per la loro obesità ma bisogna trovare un punto di equilibrio tra le responsabilità degli individui e quelle dei governi e della società.

In particolare va potenziata, secondo il Piano Sanitario Nazionale 2006-2008, e resa stabile una periodica sorveglianza dei fattori di rischio comportamentali in modo da consentire un monitoraggio delle modifiche dei comportamenti della popolazione ed una verifica di efficacia delle azioni poste in essere.

Nella regione Emilia Romagna si è avviato un sistema di Sorveglianza Nutrizionale Regionale che proseguendo l'attività iniziata nel 2003 nelle scuole materne, nell'anno 2005, ha realizzato un'indagine, in un campione rappresentativo di scuole elementari, con lo scopo di valutare lo stato nutrizionale dei bambini nati nel corso del 1996, le abitudini alimentari e gli atteggiamenti degli stessi bambini e delle loro famiglie.

Questa indagine svolta da tutte le aziende sanitarie della regione ha consentito oltre all'elaborazione dei dati a livello regionale anche una valutazione autonoma e locale dei dati in ciascuna azienda sanitaria.

Dai risultati ottenuti si potranno quindi indirizzare le politiche nutrizionali e migliorare la progettazione e la valutazione degli interventi a tutela della salute pubblica.

Dott. Luigi Salizzato

Direttore del Dipartimento di Sanità Pubblica

Introduzione

In Emilia-Romagna è in atto un progetto di sorveglianza con lo scopo di rilevare lo stato nutrizionale della popolazione infantile attraverso

- misure antropometriche (peso e altezza per calcolare l'indice di massa corporea)
- valutazione sulle abitudini alimentari.

Il piano regionale di sorveglianza nutrizionale prevede di monitorare, attraverso campioni rappresentativi, minori frequentanti le scuole materne, elementari, medie e superiori.

Nel 2003 l'indagine ha coinvolto i bambini del 3° anno della scuola materna (5-6 anni): i risultati di questo studio sono stati raccolti nel report "Sorveglianza nutrizionale nelle scuole materne dell'Azienda Sanitaria di Cesena", disponibile nel sito

http://www.ausl-cesena.emr.it/Azienda/Sanit%c3%a0Pubblica/IgieneAlimentieNutrizione/Sorveglianzanutrizionale

L'attuale indagine prende in esame i bambini iscritti al 3° anno delle scuole elementari (8-9 anni al momento della rilevazione). La scelta di questa particolare classe è motivata dal fatto che:

- diversi studi hanno evidenziato un rischio di obesità più elevato in questo gruppo di età
- evidenze scientifiche suggeriscono che l'obesità in questa fascia di età abbia un moderato valore predittivo riguardo all'obesità dell'adulto
- il bambino di 8-9 anni è sufficientemente in grado di recepire messaggi preventivi ed orientare il proprio comportamento alimentare
- la fascia di età considerata precede la pubertà, consentendo pertanto di ridurre le interferenze nella valutazione dello stato nutrizionale legate alla comparsa dello sviluppo adolescenziale, fenomeno biologico estremamente poco prevedibile e con forte variabilità interindividuale.

Obiettivi specifici a breve termine

- Stimare la prevalenza di obesità e sovrappeso nei bambini di 8-9 anni residenti nel territorio dell'Azienda Sanitaria di Cesena
- Raccogliere informazioni sulle abitudini alimentari dei bambini e dei loro genitori.

Questi dati sono fondamentali per programmare interventi sulla ristorazione scolastica (es. linee guida sulle caratteristiche dei menù scolastici), orientare iniziative di carattere formativo/informativo e di educazione sanitaria ed ideare o aggiornare percorsi di carattere assistenziale rivolti a singoli utenti a rischio.

Mirare maggiormente le scelte degli alimenti somministrati nelle mense può consentire inoltre di intervenire sul problema del rifiuto dei cibi da parte dei bambini e quindi sull'entità degli scarti.

Obiettivi specifici a medio-lungo termine

- Aumentare la diffusione di stili di vita corretti, in particolare riguardo all'alimentazione. Attivare idonei percorsi di educazione alimentare considerate le informazioni sulle reali abitudini alimentari della popolazione infantile, in collaborazione con genitori, insegnanti, direzioni didattiche, aziende di ristorazione scolastica ed operatori delle singole mense, allo scopo di ottenere i migliori risultati, in termini di efficacia, per il radicamento di stili di vita salutari.
- Aumentare la prevenzione clinica rivolta a gruppi a rischio. La migliore conoscenza dei dati sulla prevalenza di obesità e sovrappeso nella popolazione infantile, può orientare al meglio l'attività preventiva ed assistenziale di carattere individuale.

Materiali e Metodi

La popolazione oggetto di studio è costituita dai bambini nati nel 1996, e frequentanti nel 2005, il 3° anno di scuola elementare nel territorio dell'Azienda Sanitaria di Cesena, e dalle loro famiglie.

La prima fase della pianificazione dello studio ha comportato la definizione della popolazione target, del campione rappresentativo e la predisposizione dei questionari da somministrare a bambini e genitori per la raccolta di dati su consumi e comportamenti alimentari.

Nel periodo febbraio-marzo 2005 si è avviata la rilevazione preceduta da una lettera e da un incontro con le direzioni didattiche nel quale sono stati spiegati progetto ed obiettivi, consegnati i questionari diretti ai genitori e fissato il calendario per la misurazione di peso ed altezza dei bambini (previo consenso informato da parte dei genitori).

Lo studio ha coinvolto 242 bambini dei 1.568 nati nel 1996 (15%), residenti nel territorio dell'Azienda Sanitaria di Cesena e frequentanti 10 scuole del territorio.

La percentuale di famiglie che non hanno aderito all'indagine è stata inferiore al 5%.

I dati provenienti da questo studio relativi all'Azienda Sanitaria di Cesena (Sorveglianza nutrizionale 2005) sono stati confrontati con i dati regionali provenienti dalla pubblicazione "Sorveglianza nutrizionale – Determinanti e indicatori di rischio obesità nella popolazione infantile – Emilia-Romagna" e con i dati della precedente indagine aziendale svolta nelle scuole materne nell'anno 2003 (Sorveglianza nutrizionale 2003).

Metodologia del campionamento

La numerosità campionaria dei bambini è stata calcolata in modo da raggiungere un livello di precisione del 2% sulla stima del BMI. L'obiettivo del campionamento è stato l'arruolamento di almeno un numero di bambini sufficiente a garantire una rappresentatività sia a livello regionale che aziendale.

Il piano di campionamento ha previsto due stadi:

- la selezione casuale delle scuole
- l'arruolamento di tutti i bambini frequentanti la stessa classe nelle scuole selezionate.

Per evitare che le scuole di piccole dimensioni fossero sovrarappresentate, è stata applicata una procedura di campionamento che assegna ad ogni scuola un peso proporzionale al numero degli alunni delle classi in questione.

In caso di mancata collaborazione da parte di una o più scuole è stato necessario documentare le ragioni addotte per il rifiuto e sostituirla con una equivalente per tipologia, ambito territoriale e numerosità campionaria.

Indagando la composizione dei non rispondenti si è verificato che la mancata risposta non ha prodotto distorsioni di selezione.

L'elaborazione dei dati è stata condotta allo scopo di ottenere frequenze, prevalenze ed associazioni; nelle domande a risposta multipla le percentuali di ciascuna risposta sono state calcolate rispetto al numero totale delle risposte.

L'analisi statistica prevalentemente utilizzata è stata di tipo descrittivo.

Metodi e strumenti di rilevazione

Nella rilevazione delle misure antropometriche si sono seguite le modalità raccomandate dall'Organizzazione Mondiale della Sanità (OMS). Gli operatori sono stati opportunamente addestrati, al fine di minimizzare gli errori di rilevazione.

Gli alunni assenti il giorno delle misurazioni sono stati, per quanto possibile, “recuperati” successivamente.

BMI (Body Mass Index) o IMC (Indice di Massa Corporea):

E’ un indice dato dal rapporto tra il peso corporeo di un individuo (in Kg) e la sua statura al quadrato in metri.

Per la definizione di sovrappeso ed obesità nei bambini sono stati utilizzati i cut-off internazionali di BMI proposti da Cole et Al.

Valori soglia internazionali del BMI età e sesso specifici per la definizione del sovrappeso e dell’obesità in età evolutiva che intersecano il valore di BMI di 25 Kg/ m^2 e 30 kg/ m^2 a 18 anni (Cole et al. 2000)

Età (anni)	BMI 25kg/m2		BMI 30kg/m2	
	Maschi	Femmine	Maschi	Femmine
2	18,4	18,0	20,1	20,1
3	17,9	17,6	19,6	19,4
4	17,6	17,3	19,3	19,1
5	17,4	17,1	19,3	19,2
6	17,6	17,3	19,8	19,7
7	17,9	17,8	20,6	20,5
8	18,4	18,3	21,6	21,6
9	19,1	19,1	22,8	22,8
10	19,8	19,9	24,0	24,1
11	20,6	20,7	25,1	25,4
12	21,2	21,7	26,0	26,7
13	21,9	22,6	26,8	27,8
14	22,6	23,3	27,6	28,6
15	23,3	23,9	28,3	29,1
16	23,9	24,4	28,9	29,4
17	24,5	24,7	29,4	29,7
18	25,0	25,0	30,0	30,0

La classificazione utilizzata per gli adulti è quella raccomandata dall’OMS:

- “sottopeso”: persona con valori di BMI inferiori a 18,5 Kg/ m^2
- “normopeso”: persona con valori di BMI compresi nell’intervallo 18,5-24,9 Kg/ m^2
- “sovrappeso”: persona con valori di BMI compresi nell’intervallo 25-29,9 Kg/ m^2
- “obeso”: persona con valori di BMI uguali o maggiori a 30 Kg/ m^2.

Per la rilevazione sono stati utilizzati i questionari previsti dal Manuale di Sorveglianza dell'Istituto Nazionale di Ricerca per gli Alimenti e la Nutrizione (INRAN), riadattati in occasione dell'indagine 2003 sulle scuole materne e nella stessa occasione validati dal CEVEAS di Modena.

Alle famiglie i questionari sono stati consegnati dagli insegnanti, mentre quello dei bambini è stato compilato autonomamente in classe.

Il questionario indirizzato ai genitori era articolato in domande raggruppate secondo le seguenti tematiche:

- Il bambino: peso alla nascita, allattamento, eventuali restrizioni dietetiche, rapporto con il cibo
- La famiglia: peso e altezza, classe di età, grado di istruzione, professione dei genitori
- Le attività di una giornata tipo: gioco-sport
- La giornata alimentare del bambino: aspetti qualitativi, frequenze di consumo, preferenze alimentari
- Dieta e salute: fonti informative in tema di alimentazione.

Il questionario destinato ai bambini di 9 anni, conteneva domande che intendevano esplorare:

- le conoscenze dei bambini in materia di alimentazione salutare e di sani stili di vita
- le modalità con cui essi preferivano "imparare" la nutrizione
- le preferenze e le abitudini alimentari
- l'atteggiamento nei confronti del cibo
- l'immagine corporea riferita a se stessi ed ai genitori

Il questionario dei genitori, essendo un questionario autocompilato, ha presentato una percentuale di risposte non date (missing) a volte elevata (da 1,7% a 62,8%); nell'analisi sono presentati solo i dati con una percentuale di missing inferiore al 20%.

Per il questionario compilato dai bambini le percentuali di missing variano tra lo 0,3 e il 7,4% per tutte le domande tranne una, riguardante il consumo di vino che ha avuto il 47.9% di missing, probabilmente perché non pertinente alla loro età.

Per entrambi i questionari, l'anonimato è stato garantito dall'esclusivo utilizzo di un codice che consentiva, al solo rilevatore, di collegare il questionario alle misure antropometriche eseguite sul bambino.

In appendice sono riportati i questionari utilizzati.

Sintesi

Descrizione del campione

La maggior parte dei genitori è originario della nostra Regione (72%), in percentuali minori di altre regioni (18%) o dall'estero (10%).

La famiglia tipo individuata è composta da genitori che nella maggior parte dei casi hanno un'età inferiore ai 50 anni e con un livello di istruzione superiore pari al 48% nelle madri e al 40% nei padri.

In più della metà dei casi le famiglie hanno due figli.

Sovrappeso e obesità

Dalle rilevazioni condotte è risultato che la percentuale di bambini di 9 anni normopeso e sottopeso è del 70%; l'eccesso di peso è del 30% (rispettivamente 19% sovrappeso e 11% obeso). L'eccesso ponderale si distribuisce diversamente nei due sessi: tra le bambine il 27% è in sovrappeso e l'8% obesa, mentre tra i bambini le percentuali sono rispettivamente del 10% e del 15%.

L'Azienda Sanitaria di Cesena è in linea con la media regionale (70% di normopeso e sottopeso 21% di sovrappeso e 9% di obesi).

I dati regionali complessivi confermano quanto indicato in letteratura. L'indice di massa corporea dei bambini si correla all'indice di massa corporea dei genitori, in altre parole al crescere dell'indice di massa corporea della madre e del padre aumenta anche l'indice di massa corporea dei figli.

Anche il livello di istruzione dei genitori è correlato allo stato nutrizionale dei figli: con l'aumentare del livello di istruzione dei genitori, diminuisce la prevalenza di sovrappeso ed obesità dei figli.

Attività fisica

Con attività fisica si intende non solo la pratica di sport formalmente organizzati e regolarmente praticati, ma anche l'attività vivace, data dall'insieme dei movimenti del corpo che favoriscono il dispendio di energia (es. camminare di buon passo, andare in bicicletta, giocare).

Nell'indagine condotta non è risultata alcuna correlazione tra ore di attività sportiva e stato nutrizionale del bambino; invece si è rilevata correlazione tra stato nutrizionale del bambino e numero di ore di attività vivace nella giornata. Nei bambini che svolgono una maggiore attività vivace si è rilevata una minore prevalenza di soggetti sovrappeso od obesi: nei bambini che svolgono attività vivace per non più di 2 ore al giorno è sovrappeso od obeso circa il 60%, mentre la percentuale scende al 40% in chi effettua attività vivace per più di due ore al giorno.

Fra le attività sedentarie una considerazione particolare merita il problema della sempre crescente "esposizione" dei ragazzi a televisione, video-giochi e computer.

La maggior parte dei bambini ha dichiarato di passare mediamente due ore al giorno davanti alla televisione; i bambini in eccesso di peso utilizzano questo mezzo per un tempo maggiore (tre ore). Questi dati confermano l'importanza, come fattore di rischio di sovrappeso, dell'eccessivo consumo di televisione, computer, video giochi, dato già rilevato in letteratura da altri autori.

La giornata alimentare tipo

Per quanto riguarda la giornata alimentare, dall'analisi complessiva dei questionari è emersa l'abitudine a frazionare il consumo di alimenti in 5 pasti (colazione, spuntino del mattino, pranzo, spuntino pomeridiano, cena).

L'83% dei bambini di 9 anni fa colazione tutti i giorni o quasi tutti i giorni rispetto al 94% dei bambini di 6 anni.

Il latte è la bevanda maggiormente consumata (81%), seguita da succo di frutta (28%) accompagnati da prodotti da forno dolci (68%) o da fiocchi di cereali (53%) .

Il 79% dei bambini consuma uno spuntino a metà mattino composto prevalentemente da prodotti da forno dolci (54%) o salati (50%), panino imbottito (32%) accompagnati da succo di frutta o da acqua.

L'abitudine al consumo di frutta nello spuntino del mattino risultava più consistente nei bambini di 6 anni, mentre nel passaggio dai 6 ai 9 anni le abitudini/ preferenze si attestano maggiormente sui prodotti da forno dolci o salati o da panini imbottiti.

Nel 81% dei casi i bambini fanno la merenda pomeridiana tutti i giorni o quasi tutti i giorni, preferendo prodotti da forno dolci (61%), frutta (46%) o prodotti da forno salati (42%) accompagnati da yogurt o succo di frutta (55%) e da acqua (45%).

Il dopocena è consumato nel 26% dei casi, prediligendo snack dolci (43%) o frutta (39%) e bevendo acqua (54%) o latte (39%). Nei bambini di 6 anni si consumava nel 34% dei casi.

Per quanto riguarda la composizione dei pasti principali, il pranzo è composto prevalentemente dal primo, accompagnato da un secondo, contorno, frutta; a cena si conferma l'abitudine di consumare il secondo e contorno, mentre il primo viene consumato solo nel 22% dei casi. L'acqua è la bevanda più utilizzata sia a pranzo sia a cena.

Le frequenze di consumo degli alimenti

Gruppo cereali, loro derivati e tuberi

La pasta viene mangiata nel 72% dei casi una volta al giorno e il pane nel 39%. Il 52% dei bambini dichiara di mangiare patate una volta la settimana ed il 31% più volte la settimana.

Gruppo frutta e ortaggi

Solo l'11% dei bambini consuma verdura più volte al giorno, una volta al giorno il 32%; per la frutta la percentuale è simile per il consumo una volta al giorno, mentre è più elevata la quota di coloro che la consumano più volte al giorno (16%).

Gruppo latte e derivati

Il latte è bevuto una volta al giorno dai bambini nel 53% dei casi; i formaggi sono consumati nel 51% dei casi più volte a settimana e nel 17% dei casi una volta al giorno.

Gruppo carne, pesce e uova

E' più elevato il consumo settimanale di uova (57%) rispetto a quello di pesce (43%).

I salumi sono consumati nel 37% dei casi da due a quattro volte alla settimana e nel 9% una volta al giorno.

Le carni sono consumate da due a quattro volte la settimana nella maggior parte dei casi; quelle maggiormente utilizzate sono la suina, la bovina e quella di pollo. L'utilizzo di altri tipi di carne è molto minore.

I legumi sono consumati in prevalenza una volta la settimana (53%).

Il consumo di dolci (gelati, biscotti, merendine e torte) e dolciumi (caramelle, gomme o come aggiunta di zucchero negli alimenti) risulta troppo elevato: più di un terzo dei bambini li assume almeno una volta al giorno.

Le abitudini alimentari

La principale fonte di informazione in ambito alimentare è il Sistema Sanitario Nazionale che in questa fascia di età va inteso essenzialmente come pediatra di famiglia, importante figura di riferimento per genitori di bimbi ancora piccoli. Si sottolinea così l'importanza del coinvolgimento dei pediatri nella realizzazione di interventi di promozione della salute di questa fascia di età.

Dopo la nascita del figlio i genitori dichiarano di aver cambiato abitudini, aumentando il consumo di frutta e verdura (47%), acqua (36%), pesce (29%), formaggi, latte, yogurt (26%) e diminuendo il consumo di grassi/cibi grassi (48%), sale (24%); il 45% ha dichiarato di aver eliminato cibi con additivi/conservanti.

Emerge una certa sensibilità da parte dei genitori ad effettuare una corretta alimentazione; alla domanda infatti quali fattori influenzano le scelte alimentari le risposte sono state: nel 72% dei casi il tentare di mangiare sano, nel 58% "sapere cosa c'è dentro" accompagnato anche ad una tendenza a soddisfare i gusti della famiglia o all'attenzione al gusto dei cibi nel 51% e nel 40% la sicurezza alimentare.

Le difficoltà maggiormente dichiarate per effettuare una dieta più corretta sono state nel 53% i gusti della famiglia e nel 37% la vita molto impegnata e quindi poco tempo per preparare i pasti.

In particolare i maggiori ostacoli dichiarati al consumo degli alimenti considerati protettivi sono stati:

- per i legumi e le verdure il non gradimento ("non piacciono" nel 65% e nel 36% dei casi rispettivamente)
- per la frutta il fatto che non sempre è disponibile (31%)
- per il pesce il costo elevato (89%)

I bambini e la nutrizione

L'analisi dei questionari dei bambini è stata importante per comprendere il loro livello di conoscenze e ha fornito spunti interessanti per calibrare gli interventi futuri.

Sebbene infatti alla domanda "*quanto sai sulla nutrizione*?" il 74% dei bambini abbia risposto "poco" e il 6% "non so nulla", in realtà nelle domande successive riguardo a come potrebbero migliorare la loro alimentazione diminuendo o aumentando il consumo di alcuni cibi ed ancora di più sulla loro percezione dell'effetto positivo o negativo degli alimenti sullo stato di salute hanno risposto in maniera molto competente.

In particolare alle domande:

- *"Penso che dovrei mangiare di più"* più del 60% dei bambini ha risposto: pesce, frutta, minestre di verdura, frullati, legumi, verdure cotte.
- *"Penso che dovrei mangiare di meno"* più del 40% ha risposto: cioccolato, dolciumi, bevande zuccherate, patatine, gelato e dolci.

Alla domanda su come piacerebbe loro imparare la nutrizione inoltre hanno risposto poi mettendo ai primi posti i laboratori di cucina, le visite in fattoria e i programmi televisivi e agli ultimi i giornalini o i materiali informativi, privilegiando quindi il "fare" sul "conoscere".

Giornata alimentare secondo i bambini

Dall'incrocio delle risposte date sui pasti svolti con quelle che loro ritenevano giusti sono emerse queste considerazioni:

- per colazione, pranzo e cena esiste una sostanziale correlazione tra quello che fanno e quello che ritengono giusto
- per lo spuntino della mattina e del pomeriggio il 25-30% dei bambini non lo ritiene giusto anche se lo fa, non riconoscendo l'importanza nutrizionale di interrompere il digiuno tra il pranzo e la cena
- per il dopocena o i fuori pasto c'è una consapevolezza della non correttezza di questi pasti e solo l'8% e il 6% rispettivamente lo ritiene giusto.

Sei d'accordo o no

Per quanto riguarda il tema alimentare hanno opinioni ben consolidate su alcune tematiche ormai oggetto di numerose campagne di informazione.

Ti piace

E' stato sondato sia il gradimento dei bambini per alcuni alimenti sia se siano mai stati assaggiati.

Il 33% dei bambini dichiara di non aver mai assaggiato i broccoli, il 27% la frutta esotica ed il 22% i carciofi.

Fra le verdure il 78% dei bambini ha dichiarato di amare molto le carote, il 65% i finocchi, il 58% pomodori e insalata, mentre non piacciono i i broccoli nel 57%, il cavolfiore nel 52% e i carciofi nel 51%.

Tra i pesci piacciono molto tonno (71% dei casi) e i bastoncini (69%).

Igiene orale

La carie dentale è la patologia dei denti più diffusa; è una malattia per cui gli acidi prodotti dai batteri dissolvono i denti con un processo di demineralizzazione.

Al processo di remineralizzazione contribuisce la saliva, pulendo i residui di cibo dalla bocca, neutralizzando gli acidi prodotti dai batteri, fornendo calcio e fosfato ai denti e il fluoro inibendo la produzione di acido.

La carie dentaria si verifica soltanto quando il processo di demineralizzazione è più intenso di quello di remineralizzazione nell'arco di un certo periodo di tempo.

I seguenti fattori hanno conseguenze importanti per la salute dentale:

- **fattori individuali** (forma della mandibola e della cavità orale, struttura dei denti, quantità e qualità della saliva)
- **igiene orale e uso di fluoro**: si ritiene che la pulizia dei denti con dentifricio al fluoro sia il fattore più importante nel calo della carie dentaria osservato in molti Paesi. L'uso dello spazzolino e del filo interdentale, abbinato alla contemporanea applicazione di fluoro, aiuta ad eliminare i batteri dalla bocca riducendo il rischio di carie. I controlli dentistici regolari possono contribuire a rilevare e tenere sotto controllo i potenziali problemi.
- **fattori alimentari**: le abitudini alimentari, le caratteristiche degli alimenti e la frequenza del consumo continuano a influire sul rischio di insorgenza di questa malattia.

Dal nostro studio emerge che il 55% dei bambini si lava i denti dopo i pasti principali, nel 92% dei casi usa dentifricio, solo il 18% il filo interdentale e solo il 19% spazzola i denti per più di due minuti.

Descrizione del campione

La famiglia

All’interno del contesto di vita, la famiglia ha certamente un’influenza determinante nell’apprendimento e nell’acquisizione di stili di vita e comportamenti che contribuiscono e contribuiranno a definire la salute dei giovani e futuri adulti. In particolare si è ravvisata una correlazione tra lo stato socio-economico e il livello culturale delle famiglie e lo stato nutrizionale dei bambini.

La maggior parte dei genitori è originario della nostra Regione (72%), in percentuali minori di altre regioni (18%) o dall’estero (10%). Il dato rilevato per i bambini delle materne nell’indagine precedente mostra una minor presenza di genitori provenienti dall’estero (5%).

Provenienza.
Confronto tra i bambini di 6 e 9 anni

Genitori dei bambini di 6 anni
(Sorveglianza nutrizionale anno 2003)

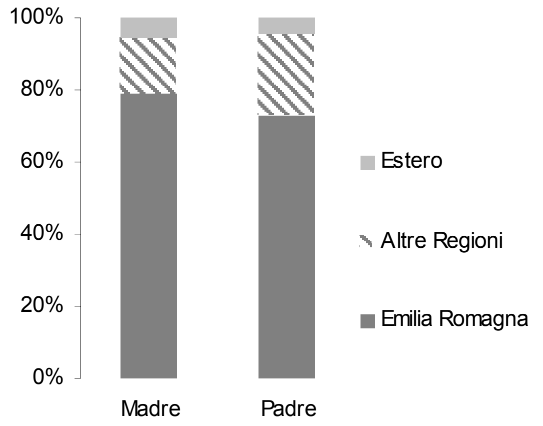

Genitori dei bambini di 9 anni
(Sorveglianza nutrizionale anno 2005)

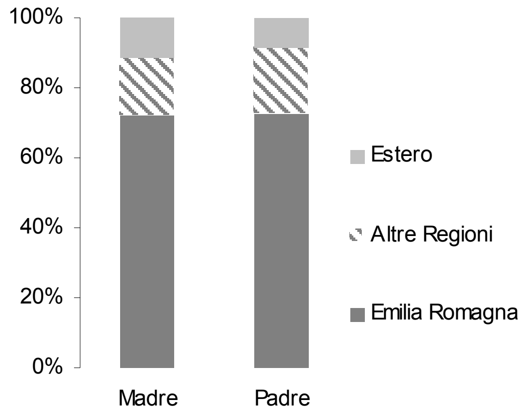

La famiglia tipo individuata è composta da genitori che nella maggior parte dei casi hanno un’età inferiore ai 50 anni e con un livello di istruzione superiore pari al 48% nelle madri e al 40% nei padri.

In più della metà dei casi le famiglie hanno due figli.

Il titolo di studio
(Sorveglianza nutrizionale, anno 2005)

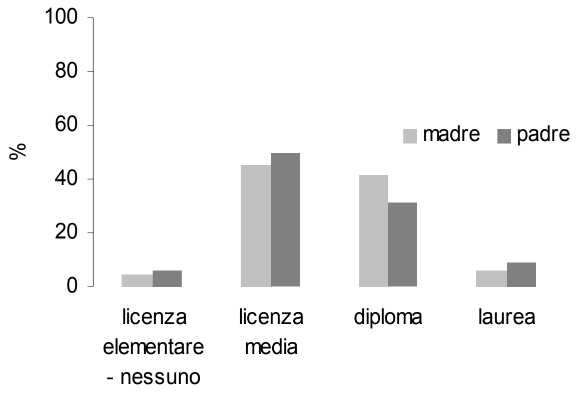

L'età
(Sorveglianza nutrizionale, anno 2005)

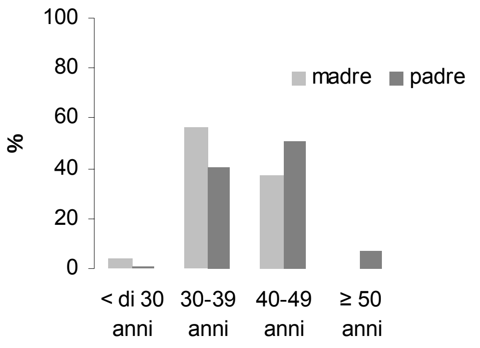

L'analisi delle professioni svolte dai familiari dei bambini evidenzia un livello socio-economico mediamente elevato. In molte famiglie lavorano entrambi i genitori; in poco più di un quarto dei casi le madri svolgono attività di casalinga; praticamente nulla è la percentuale di genitori in cerca di occupazione.

La professione
(Sorveglianza nutrizionale, anno 2005)

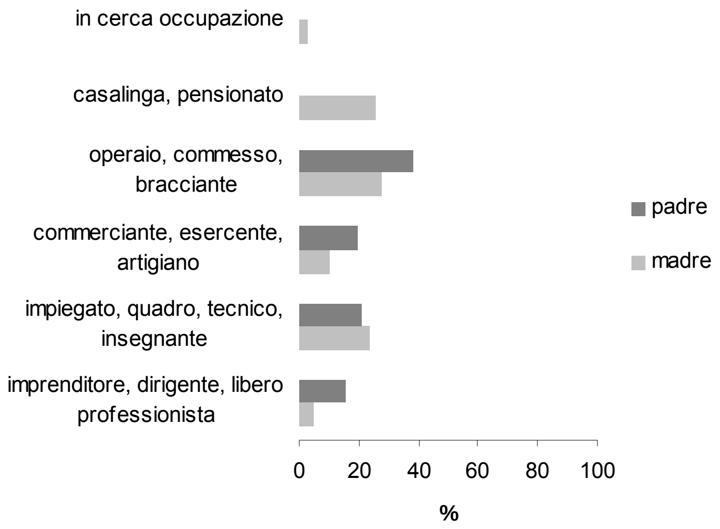

I bambini

Lo studio ha coinvolto 242 bambini sulla popolazione di circa 1.568 bambini nati nel 1996 residenti nel territorio dell'Azienda Sanitaria di Cesena. Le scuole selezionate sono state 10.

Il campione era composto per il 48% di bambini e per il 52% di bambine.

Nella storia alimentare del bambino, si trova il 69% di allattati al seno. Il latte materno con la ricchezza dei suoi componenti costituisce un vero e proprio "sistema biologico" che si associa non solo a migliori parametri di crescita, ma anche ad un migliore sviluppo neuro-comportamentale e alla prevenzione di varie patologie acute e croniche. Per tale motivo l'OMS definisce “l'allattamento al seno esclusivo” l'alimentazione ideale nei primi sei mesi di vita.

Dalle evidenze disponibili in letteratura l'allattamento materno, specialmente se prolungato, sembra anche avere un ruolo protettivo nei confronti dell'obesità; è però necessaria una rassegna sistematica, che comprenda studi di grandi dimensioni, per valutare l'effetto dei fattori confondenti in maniera più dettagliata. Esistono, invece, evidenze ben consolidate sui benefici, a breve e a lungo termine, dell'allattamento al seno, quali una migliore crescita nel bambino anche pretermine, un miglior sviluppo neurale e psico-sociale, una riduzione delle malattie allergiche.

Da alcune indagini risulta, infatti, che circa il 3-5% della popolazione scolastica necessita di regimi dietetici particolari: l'85% di questi è affetto da allergie o intolleranze alimentari ed il restante 15% richiede una dieta differenziata come parte integrante della terapia per malattie croniche.

In caso di allergie può essere sufficiente una piccola quantità dell'alimento a scatenare la sintomatologia allergica, quindi è indispensabile porre particolare attenzione nella formulazione della dieta per escludere completamente gli alimenti in causa. L'allergia alimentare è una forma di esagerata sensibilità a sostanze inalate, ingerite, iniettate o che vengono a contatto con pelle e occhi; essa è una malattia del sistema immunitario, che normalmente ci difende dalle aggressioni esterne.

Le intolleranze alimentari invece non sono mediate da meccanismi immunologici, possono dipendere da un difetto enzimatico o dall'azione, in individui predisposti, di alcune sostanze con un attività di tipo farmacologico presenti negli alimenti.

Nel nostro studio è risultata la presenza di allergie testate clinicamente nel 2% dei casi e intolleranze alimentari nel 3%.

Sovrappeso ed obesità

Sovrappeso ed obesità nei bambini e nei giovani

Secondo le attuali conoscenze scientifiche, l'obesità, un'alimentazione non corretta ed errori dietetici sono un importante fattore di rischio per la salute dell'individuo e sono in stretta correlazione con numerose malattie.

Le condizioni dei bambini e dei ragazzi destano le maggiori preoccupazioni perché l'obesità infantile è il principale fattore di rischio per lo sviluppo dell'obesità da adulti.

Il rischio relativo per un bambino obeso di diventare un adulto obeso aumenta con l'età ed è direttamente proporzionale alla gravità dell'eccesso ponderale. Fra i bambini obesi in età prescolare, dal 26 al 41% è obeso da adulto, e fra i bambini in età scolare tale percentuale sale al 69%. Complessivamente il rischio per i bambini obesi di divenirlo da adulti è da 2 a 6 volte maggiore rispetto ai bambini non obesi.

Diversi fattori sono coinvolti nel favorire il mantenimento di uno stato di obesità. In ordine temporale, i fattori familiari (siano essi genetici o legati a variabili ambientali) possono senza dubbio condizionare uno stato di obesità infantile, che avrà maggiori possibilità di sfociare in un'obesità permanente quanto maggiore è il grado di obesità e la sua durata. Altri fattori come la scarsa attività fisica, il frequente weight cycling (oscillazioni ponderali ripetute), la mancata adesione a corretti protocolli di follow-up possono, a loro volta, contribuire al mantenimento di uno stato di obesità anche in età adulta. Il futuro che si presenta è quello di una nuova generazione, nella quale la malattia sarà sempre più diffusa.

Il sovrappeso, l'obesità e le relative conseguenze, inoltre non sono distribuite uniformemente nella popolazione. Come per altre condizioni di vita e di salute, si osserva una grande variabilità di carattere geografico, associata a disuguaglianze sociali.

Situazione internazionale

In tutta L'Europa e in quasi tutti i paesi occidentali l'obesità ha assunto i connotati di una vera e propria epidemia come è stata definita nel rapporto sulla salute in Europa (OMS, European Health report 2002). Nel corso degli ultimi decenni, negli USA la prevalenza dell'obesità nell'adulto e nel bambino è pressoché raddoppiata, crescendo con una rapidità tale che, se si mantenesse inalterata, porterebbe, nei prossimi decenni, la quasi totalità della popolazione americana ad essere obesa.

In Brasile, unico paese del Sud-America ad avere attivato negli ultimi 10 anni un sistema di sorveglianza nutrizionale, l'obesità è in aumento specialmente tra i gruppi di popolazione a basso reddito. In effetti, la cosiddetta "globalizzazione" ha cambiato il volto dell'obesità in vari paesi. Nello stesso Brasile ed in Messico, ove il sovrappeso è sempre stato un simbolo di ricchezza, ora tale condizione si associa frequentemente ad uno stato di povertà. Ciò perché, mentre la popolazione di fascia economico-sociale più alta è maggiormente sensibilizzata all'adozione di stili di vita salutari, i poveri hanno sia una ridotta possibilità di scelta in campo alimentare, sia un accesso limitato a programmi di educazione nutrizionale.

Scarsi sono i dati per quanto riguarda le popolazioni Medio Orientali, quelli disponibili sembrano indicare come la prevalenza dell'obesità in queste regioni sia alta, particolarmente nelle donne. Per queste ultime si segnalano spesso indici superiori a quelli delle donne residenti nella maggior parte dei paesi occidentali.

Anche in Cina l'obesità è in aumento in particolare, nelle aree urbane e, ancora, nel sesso femminile.

In Giappone come in altre aree del Pacifico (Micronesia e Polinesia) la prevalenza dell'obesità è drammaticamente aumentata negli ultimi 20 anni.

Situazione in Italia

Secondo i risultati dell'indagine Multiscopo dell'Istat, in Italia i bambini di 6-9 anni hanno una percentuale di eccesso di peso del 34% nei maschi e del 35% nelle femmine; nei ragazzi di 6-17 anni l'eccesso di peso è stimato nel 24% dei casi. Si osserva inoltre una crescita dell'obesità infantile passando dal nord al sud del paese.

Il Ministero della Salute, nel Piano Sanitario Nazionale 2006-2008, ribadisce l'efficacia della riduzione dei fattori di rischio nel prevenire patologie legate all'alimentazione. Ai fini della prevenzione dell'obesità indica come rilevanti, oltre a quelle previste dal Piano Nazionale della Prevenzione, l'attuazioni di politiche:

- atte a riorientare la produzione alimentare
- regolamentare in modo più puntuale le informazioni contenute nella pubblicità
- coinvolgere maggiormente gli operatori sanitari perchè svolgano azioni concrete nel campo della corretta informazione e dell'educazione sanitaria.

Il Piano Nazionale della Prevenzione 2005-2007 individua l'obesità, in particolare nelle donne in età fertile e nei bambini, come problema di salute prioritario.

Il Centro Nazionale per la Prevenzione e il Controllo delle Malattie (CCM) ha messo a punto una strategia generale che mira da un lato a disporre di informazioni affidabili su obesità e sovrappeso, pattern nutrizionali e di attività fisica della popolazione italiana in età infantile ed adulta, dall'altro a permettere la pianificazione, a livello regionale e locale, di interventi volti a migliorare la nutrizione ed a promuovere l'attività fisica presso la popolazione.

Situazione locale

Nel Piano Regionale della Prevenzione, l'Emilia-Romagna ha inserito la sorveglianza e prevenzione dell'obesità tra gli obiettivi 2006-2008. La strategia operativa del piano è quella di creare una rete nel cui ambito varie istituzioni, a livello regionale e locale, cooperano per promuovere un contesto che renda agevoli le scelte nutrizionali sane e lo svolgimento di una regolare attività fisica.

Dalle rilevazioni condotte nel presente studio è risultato che il 70% dei bambini di 9 anni è sottopeso o normopeso, mentre il 30% presenta un eccesso ponderale (19% sovrappeso e 11% obeso). L'Azienda Sanitaria di Cesena è in linea con la media regionale (rispettivamente 70%, 21% e 9%). L'eccesso di peso si distribuisce diversamente nei due sessi: nel cesenate il 27% delle bambine è in sovrappeso e l'8% è obesa, mentre nei bambini i valori sono pari al 10% e al 15% (in Regione 20% delle bambine è in sovrappeso e l'8,4% è obesa mentre nei bambini il 21,3% è in sovrappeso e il 9,5% è obeso).

Stato nutrizionale
(Sorveglianza nutrizionale, anno 2005)

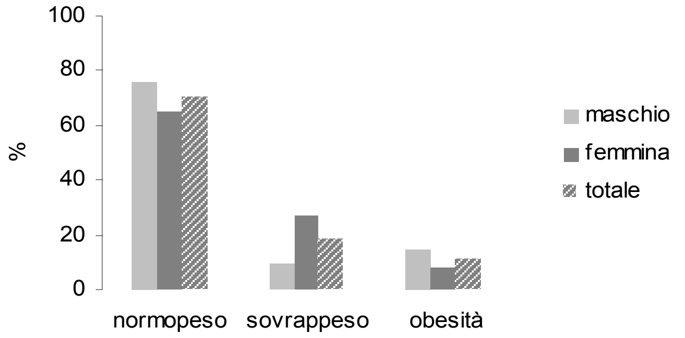

L'indice di massa corporea dei bambini si correla all'indice di massa corporea dei genitori in linea con i dati di letteratura: al crescere dell'indice di massa corporea della madre e del padre aumenta anche l'indice di massa corporea dei figli.

Anche il livello di istruzione dei genitori, associato spesso allo stato socio-economico, è correlato allo stato nutrizionale dei figli: con l'aumentare del livello di istruzione dei genitori, diminuisce la prevalenza di sovrappeso ed obesità dei figli.

Studi condotti sulla popolazione, in effetti, mostrano nette differenze tra i vari ceti sociali in relazione all'assunzione di cibo e nutrienti. I gruppi a basso reddito, in particolare, hanno una maggior tendenza a seguire una dieta sbilanciata ed evidenziano un minor consumo di frutta e verdura.

Questo determina sia malnutrizione (carenza di micronutrienti) sia sovralimentazione (eccessivo consumo calorico che genera sovrappeso e obesità) tra i membri di una comunità, a seconda della fascia di età, del sesso e del livello di privazione. I meno favoriti sviluppano anche malattie croniche in età più giovane rispetto ai gruppi socio-economici più elevati, di solito identificati in base al livello di istruzione e di occupazione.

Le cause di molti dei problemi che le persone con un reddito basso devono affrontare sottolineano la necessità di un approccio multidisciplinare volto a individuare le esigenze sociali e a migliorare le ineguaglianze nel campo della salute. I fattori che influenzano la scelta degli alimenti non si basano soltanto su preferenze individuali, ma sono limitati da circostanze sociali, culturali ed economiche. I gruppi a basso reddito devono far fronte a specifiche difficoltà quando cercano di modificare la propria alimentazione, e le soluzioni devono essere altrettanto specifiche e mirate.

Attività fisica

Modelli di vita che prevedono una sufficiente pratica quotidiana di attività fisica presentano molteplici effetti positivi: un corretto sviluppo fisico, un'importante prevenzione rispetto al rischio di sovrappeso e obesità, ricadute positive in termini psicologici (autostima, soddisfazione di sé, sviluppo della socialità), nonchè l'acquisizione di un modello comportamentale attivo che aumenti le probabilità di una vita adulta equilibrata.

Con attività fisica non si intende solo la pratica di sport formalmente organizzati e regolarmente praticati, ma l'insieme dei movimenti del corpo che favoriscono il dispendio di energia (camminare di buon passo, andare in bicicletta, giocare,…).

Esiste invece una netta tendenza, nelle popolazioni dei paesi occidentali, a trascorrere il tempo libero in attività sedentarie, riducendo al minimo le attività di movimento. La sedentarietà è diventata, progressivamente negli ultimi decenni, una caratteristica dominante dello stile di vita di una parte, purtroppo sempre maggiore, della nostra popolazione.

In tal senso, è stato anche possibile verificare che, tra le nuove generazioni, sono quasi del tutto sconosciuti i giochi tradizionali che in passato impegnavano gran parte del tempo libero dei giovani.

Attività fisica in Italia

Secondo i dati ISTAT 2003, nella fascia di età 6 -10 anni aumentano i comportamenti sedentari: quasi un bambino su quattro (24%) non svolge alcuna attività fisica nel tempo libero con una differenza tra femmine (27%) e maschi (22%). In particolare diminuisce la quota di quanti svolgevano almeno attività fisica di tipo destrutturato (passeggiate in bicicletta, a piedi…), solo parzialmente compensata da lievi incrementi dell'attività sportiva, sia continuativa che saltuaria.

Attività fisica nella Regione Emilia-Romagna

L'analisi dell'attività motoria dei bambini, nel nostro studio è stata effettuata chiedendo ai genitori di suddividere la giornata standard dei loro figli in base al tempo dedicato a riposo, scuola, attività vivace e attività leggera. Per attività leggera si è inteso il tempo trascorso a leggere, mangiare, lavarsi, guardare la TV/videogiochi, svolgere giochi sedentari. Nell'attività vivace è stata inclusa tutta l'attività motoria con significativo (anche se variabile) dispendio energetico, svolta in orario extra-scolastico. Il tempo trascorso a scuola è stato considerato nel suo complesso, non essendo possibile suddividerlo a seconda dell'impegno fisico.

Un terzo (33%) dei bambini di 9 anni non svolge alcuna attività sportiva. Tra coloro che la praticano, gli sport preferiti sono il nuoto, il calcio, la danza e la ginnastica artistica.

Nell'indagine condotta non è risultata alcuna correlazione tra ore di attività sportiva e stato nutrizionale del bambino; invece si è rilevata correlazione tra stato nutrizionale del bambino e numero di ore di attività vivace nella giornata. Nei bambini che svolgono una maggiore attività vivace si è rilevata una minore prevalenza di soggetti sovrappeso od obesi: nei bambini che svolgono attività vivace per non più di 2 ore è sovrappeso od obeso circa il 60%, mentre la percentuale scende al 40% in chi effettua attività vivace per più di due ore al giorno.

Fra le attività sedentarie una considerazione particolare merita il problema della sempre crescente "esposizione" dei ragazzi alla televisione, a video-giochi e computer.
E' risultata ancora una correlazione tra numero di ore davanti alla televisione e stato nutrizionale del bambino di 9 anni.
Se nella maggior parte dei bambini sono state dichiarate mediamente due ore giornaliere di televisione, si sottolinea come siano più spesso i bambini in eccesso di peso ad usufruire di questo mezzo per un tempo maggiore. Questi dati confermano, anche nella nostra popolazione l'importanza come fattore di rischio di obesità e sovrappeso, dell'eccessivo consumo di televisione, computer, video giochi, già rilevato in letteratura da altri autori.

La giornata alimentare tipo

Le abitudini alimentari, che influiscono sulle preferenze in fatto di cibi, sul consumo energetico e sull’assunzione di nutrienti, si sviluppano generalmente nella prima infanzia e, in particolare, durante l’adolescenza. L’ambiente domestico e quello scolastico svolgono un ruolo essenziale nella definizione del rapporto del bambino col cibo e del consumo dei singoli alimenti.

In particolare gli adolescenti, esposti a mode alimentari periodiche e ai trend della magrezza a tutti i costi, sono portati a saltare i pasti e a sviluppare abitudini alimentari irregolari. Uno dei pasti più spesso trascurati è la colazione. Vari studi dimostrano che la colazione è invece fondamentale in quanto fornisce energia e nutrienti dopo il digiuno notturno e può contribuire al miglioramento della concentrazione e del rendimento scolastico.

Anche gli spuntini sono generalmente parte integrante dei regimi alimentari consigliati per bambini e adolescenti. I bambini più piccoli non possono mangiare grandi quantità di cibo in una sola volta e spesso hanno fame molto prima dell’orario previsto per il pasto successivo. Lo spuntino di metà mattina e la merenda possono aiutare a soddisfare il fabbisogno energetico per tutto il giorno.

Per quanto riguarda la giornata alimentare tipo, emerge l’abitudine a frazionare il consumo di alimenti in 5 pasti (colazione, spuntino del mattino, pranzo, spuntino pomeridiano, cena).

L'83% dei bambini di 9 anni fa colazione tutti i giorni o quasi tutti i giorni, rispetto al 94% del bambini di 6 anni (Sorveglianza nutrizionale anno 2003). Inizia a delinearsi una tendenza alla diminuzione del consumo della prima colazione che in genere risulta più marcata nelle età successive, rilevata anche a livello regionale.

La bevanda maggiormente consumata è il latte (81%), seguita da succo di frutta (28%) e accompagnata da prodotti da forno dolci (68%) o da fiocchi di cereali (53%).

Alimenti assunti a colazione.
Confronto tra i bambini di 6 e 9 anni

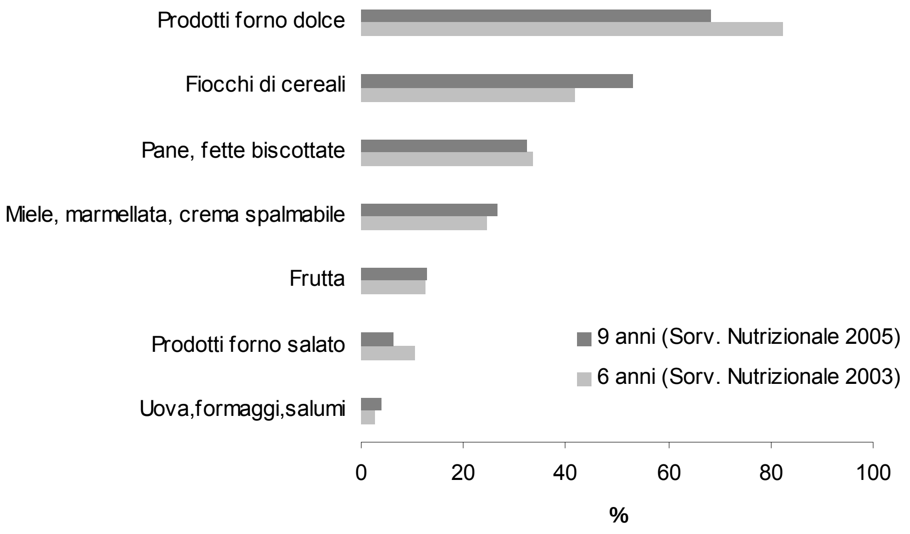

Bevande assunte a colazione.
Confronto tra i bambini di 6 e 9 anni

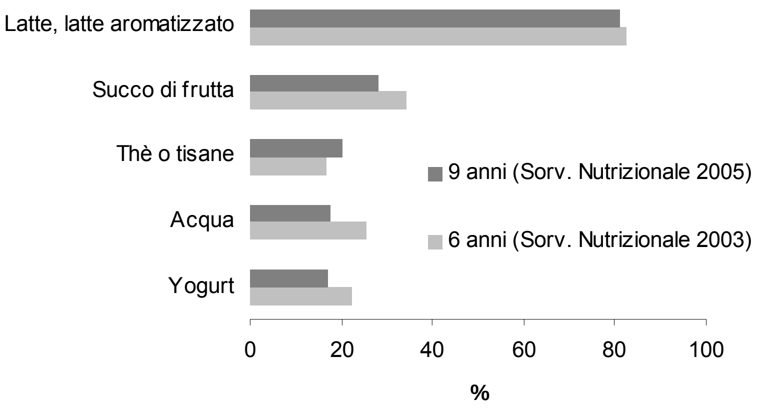

Lo spuntino a metà mattino è composto prevalentemente da prodotti da forno dolci (54%) o salati (50%) e dal panino imbottito (32%), accompagnati per lo più da succo di frutta e acqua.

L'abitudine al consumo di frutta nello spuntino del mattino risulta più consistente nei bambini di 6 anni mentre nel passaggio dai 6 ai 9 anni le abitudini/ preferenze si attestano maggiormente sui prodotti da forno dolci o salati o sui panini.

Alimenti assunti nello spuntino del mattino.
Confronto tra i bambini di 6 e 9 anni

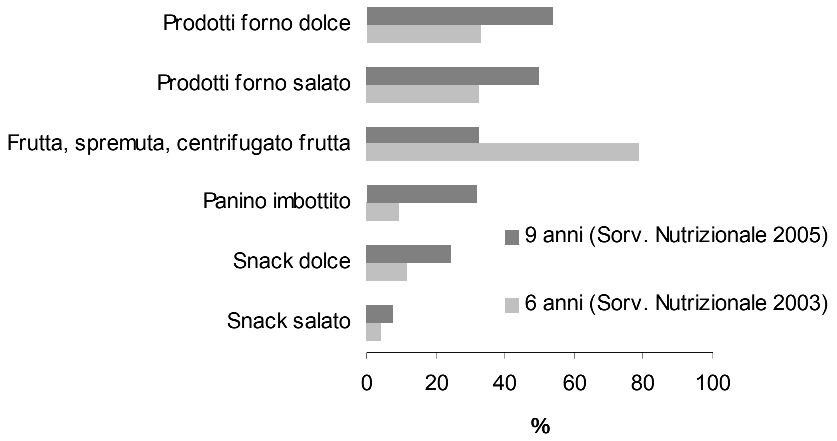

Bevande assunte nello spuntino del mattino.
Confronto tra i bambini di 6 e 9 anni

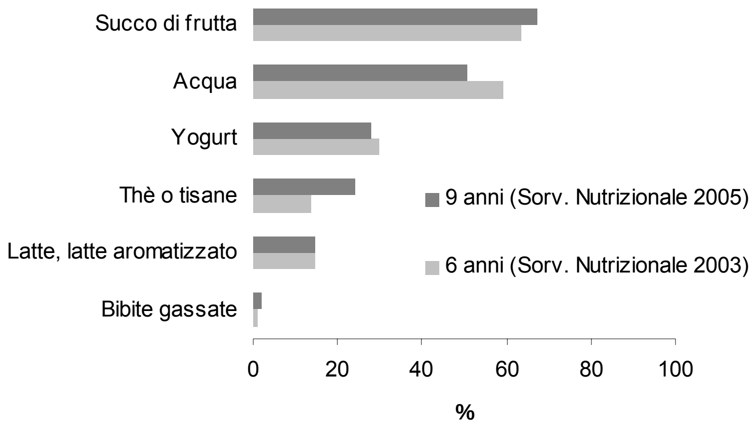

La merenda pomeridiana viene fatta preferendo prodotti da forno dolci (61%), frutta (46%) o prodotti da forno salati (42%) accompagnati da yogurt o succo di frutta (55%) e da acqua (45%).

Il succo di frutta e l'acqua erano utilizzati in misura maggiore nei bambini di 6 anni.

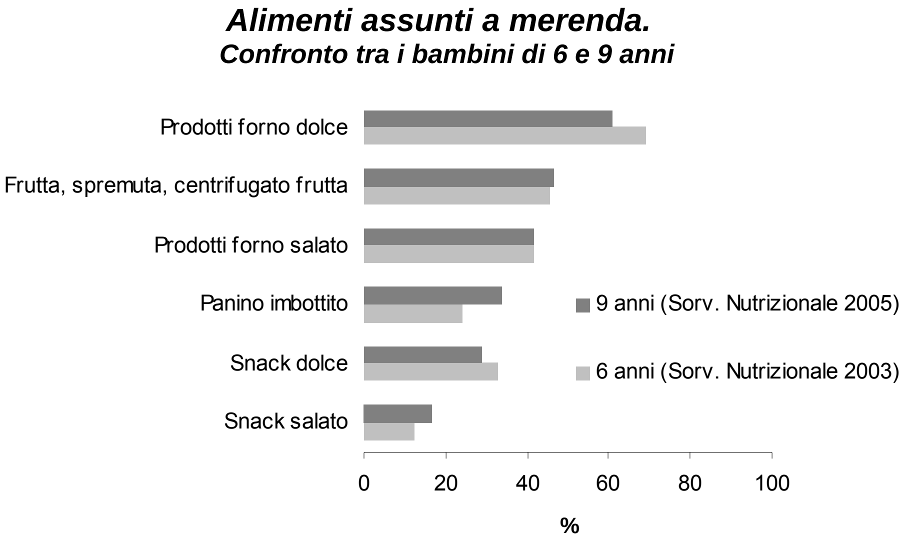

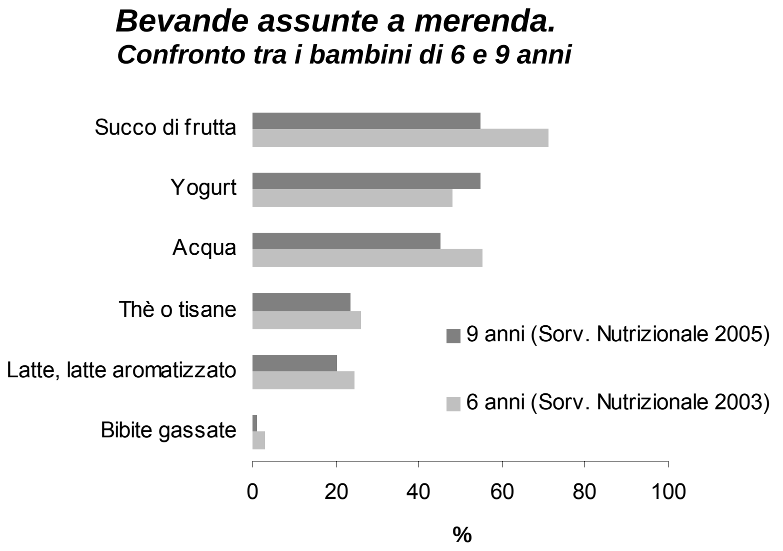

Il dopocena è consumato nel 26% dei casi (vs 20% del dato regionale) prediligendo snack dolci (43%) o frutta (39%) e bevendo acqua (54%) o latte (39%).

Questa percentuale è inferiore a quella rilevata nei bambini di 6 anni (34% vs 27% del dato regionale).

Alimenti assunti nel dopocena.
Confronto tra i bambini di 6 e 9 anni

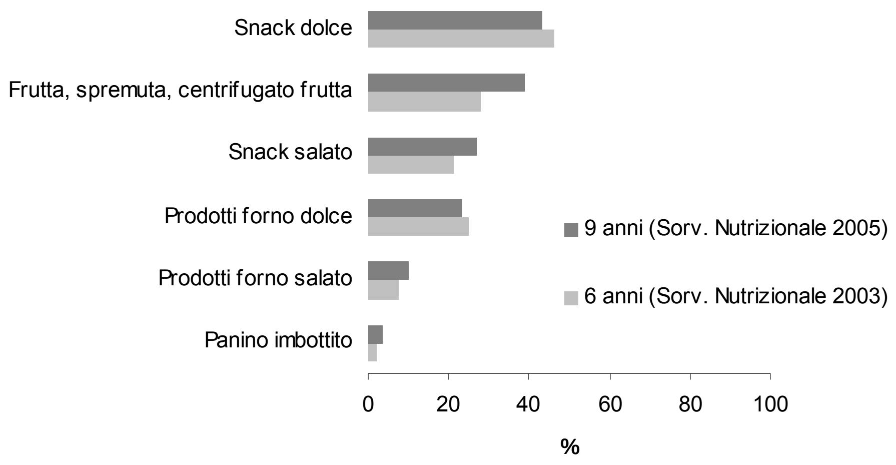

Bevande assunte nel dopocena.
Confronto tra i bambini di 6 e 9 anni

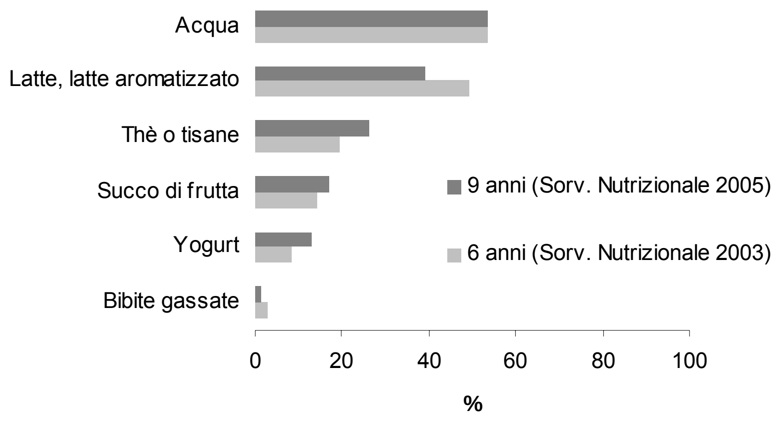

Per quanto riguarda la composizione dei pasti principali, il pranzo è composto prevalentemente da primo, secondo, contorno e frutta; a cena si conferma l'abitudine di consumare secondo e contorno, mentre il primo viene consumato solo nel 22% dei casi. L'acqua è la bevanda più utilizzata sia a pranzo sia a cena.

Rispetto ai bambini di 6 anni, quelli di 9 sono meno abituati a consumare il secondo e il contorno durante il pranzo.

Alimenti assunti a pranzo.
Confronto tra i bambini di 6 e 9 anni

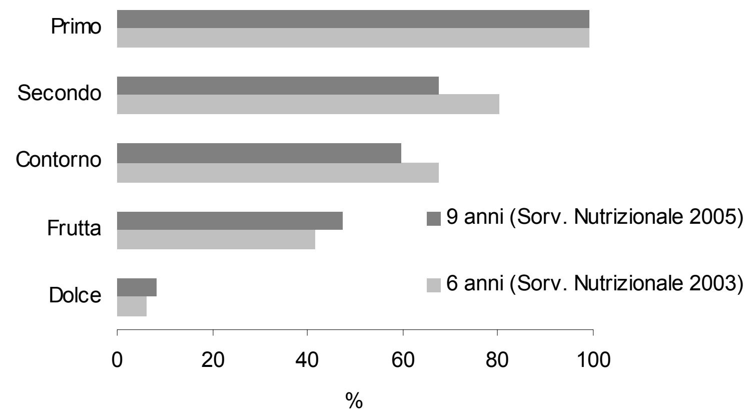

Bevande assunte a pranzo.
Confronto tra i bambini di 6 e 9 anni

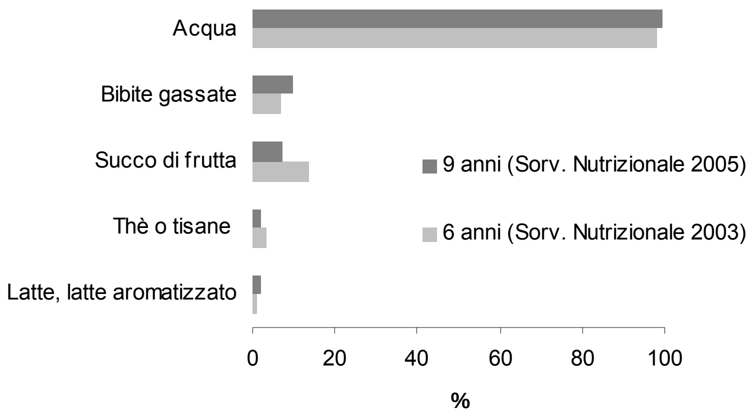

Alimenti assunti a cena.
Confronto tra i bambini di 6 e 9 anni

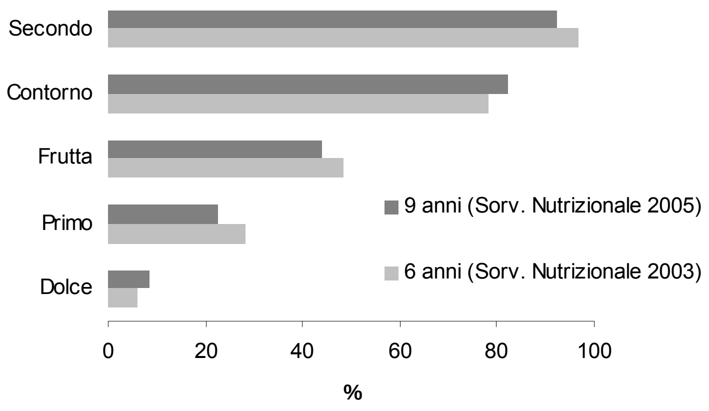

Bevande assunte a cena.
Confronto tra i bambini di 6 e 9 anni

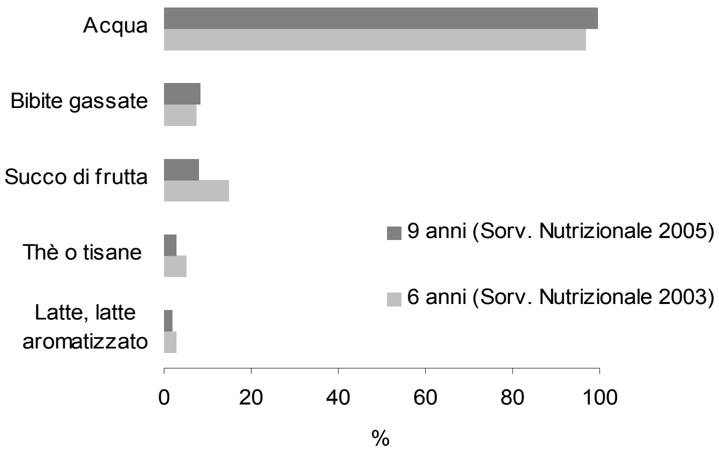

Complessivamente le preferenze alimentari dei bambini della nostra azienda sono in linea con quelle regionali.

Le frequenze di consumo degli alimenti

Il gruppo **cereali, loro derivati e tuberi** comprende: pane, pasta, riso, altri cereali minori oltre alle patate. Essi apportano buone quantità di vitamine del complesso B e proteine che, unite a quelle dei legumi, possono costituire una miscela proteica di valore biologico paragonabile a quello delle proteine animali. Le porzioni consigliate sono: tre di pane, una di prodotti da forno, una di pasta al giorno e una porzione di patate alla settimana.

La pasta viene consumata una volta al giorno dal 72% dei bambini, il pane una volta al giorno dal 39% e più volte al giorno dal 33%.

Il 52% mangia patate una volta la settimana, il 31% più volte la settimana ed il 7% una volta al giorno.

Per quanto riguarda il pane e la pasta, la loro assunzione è da ritenersi per lo più corretta. Il consumo di patate risulta invece eccessivo, in quanto in più di un terzo dei casi vengono mangiate più volte a settimana.

Frequenza di consumo dei cereali
(Sorveglianza nutrizionale anno 2005)

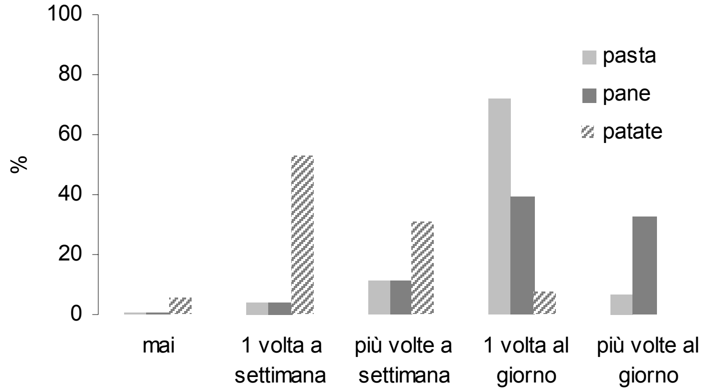

Il gruppo costituito da **frutta ed ortaggi**, comprendenti anche i legumi freschi, rappresenta una fonte importantissima di fibra, vitamine e sali minerali; se ne consigliano cinque porzioni al giorno.

Il 32% dei bambini consuma verdura almeno una volta al giorno, ma solo l'11% la consuma più volte al giorno. Per la frutta la percentuale di chi la consuma almeno una volta al giorno è simile (33%) a quella relativa al consumo di verdura, mentre è più elevata la quota di coloro che la consumano più volte al giorno (16%).

Frequenza di consumo di frutta e verdure
(Sorveglianza nutrizionale anno 2005)

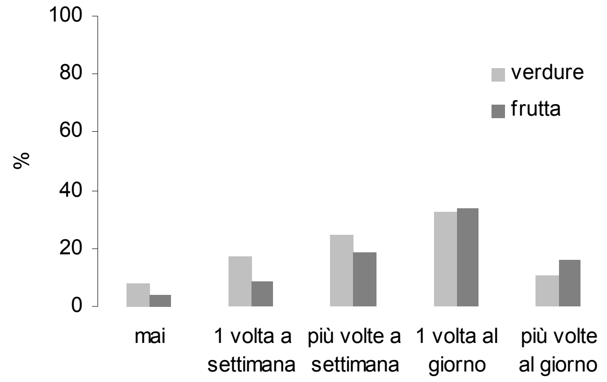

Il gruppo **latte e derivati** comprende il latte, lo yogurt e i formaggi; la loro funzione principale è fornire calcio, in forma altamente biodisponibile, ossia facilmente assorbibile e utilizzabile. Gli alimenti di questo gruppo contengono inoltre proteine di ottima qualità biologica ed alcune vitamine. Le porzioni consigliate di latte e yogurt sono di tre al giorno, mentre per i formaggi una o due alla settimana.

Il latte viene bevuto una volta al giorno dal 53% dei bambini, più volte al giorno dal 14%.

I formaggi sono consumati nel 17% dei casi una volta al giorno, nel 30% una volta alla settimana, nel 13% mai. Nonostante il 30% circa dei bambini assuma formaggio nella quantità raccomandata, è elevata la percentuale di coloro che ne mangiano troppo (51% più di una volta a settimana) o troppo poco (13%).

Frequenza di consumo di latte e formaggi
(Sorveglianza nutrizionale anno 2005)

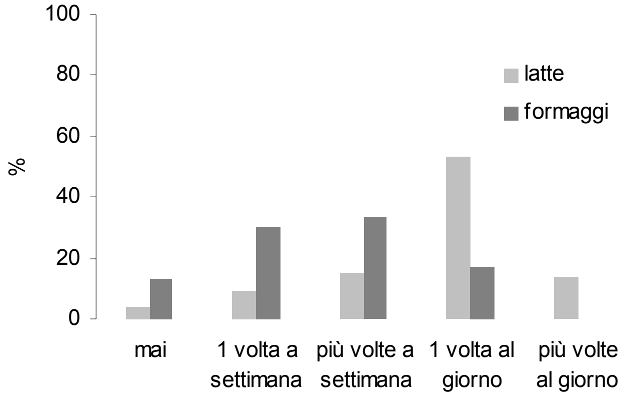

Il gruppo **carne, pesce e uova** ha la funzione principale di fornire proteine di elevata qualità, oligoelementi e vitamine del complesso B. Nell'ambito del gruppo sono da preferire le carni magre e il pesce; va invece moderato, per quanto riguarda la quantità, il consumo di prodotti a maggiore tenore in grassi, quali certi tipi di carne e di insaccati. In questo gruppo è conveniente da un punto di vista nutrizionale includere i legumi secchi: questi, oltre a rilevanti quantità di amido e di fibra forniscono ferro, altri oligoelementi e notevoli quantità di proteine di buona qualità biologica. Per carne, pesce e legumi le porzioni consigliate sono di 3-4 porzioni alla settimana per ognuno; per le uova una o due alla settimana.

Il consumo di uova risulta più elevato rispetto a quello di pesce (57% vs 43% una volta a settimana).

I salumi sono consumati dal 41% dei bambini una volta alla settimana, dal 37% da due a quattro volte alla settimana, dal 9% una volta al giorno.

I legumi sono consumati in prevalenza una volta la settimana (53%).

Le carni sono consumate dalla maggior parte da due a quattro volte la settimana; quelle più utilizzate sono la suina, la bovina e quella di pollo. L'utilizzo di altri tipi di carne è molto minore.

Il consumo di **dolci** (gelati, biscotti, merendine e torte) e **dolciumi** (caramelle, gomme o come aggiunta di zucchero negli alimenti) risulta troppo elevato: più di un terzo dei bambini li assume almeno una volta al giorno.

Frequenza di consumo di dolci e dolciumi
(Sorveglianza nutrizionale anno 2005)

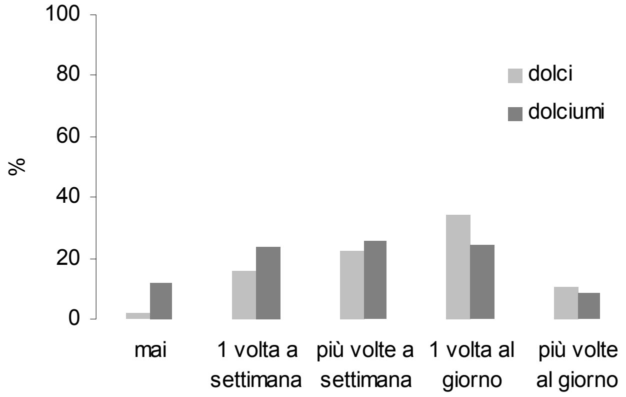

Le abitudini alimentari

Fonti di informazione sull'alimentazione

La famiglia è attenta a più fonti di informazione nutrizionale: la principale fonte di informazione in ambito alimentare è rappresentata dal Sistema Sanitario Nazionale, che in questa fascia di età va inteso essenzialmente come pediatra di famiglia, importante figura di riferimento che segue con continuità fin dalla nascita il bambino e i suoi genitori.

Poiché una discreta percentuale di genitori ha un titolo di studio medio-alto, non stupisce il fatto che altre fonti di informazione siano rappresentate da libri o testi specializzati, e vi sia attenzione anche alla lettura delle etichette; la scuola è individuata quale fonte informativa da un terzo circa delle famiglie.

Fonti di informazione
(Sorveglianza nutrizionale anno 2005)

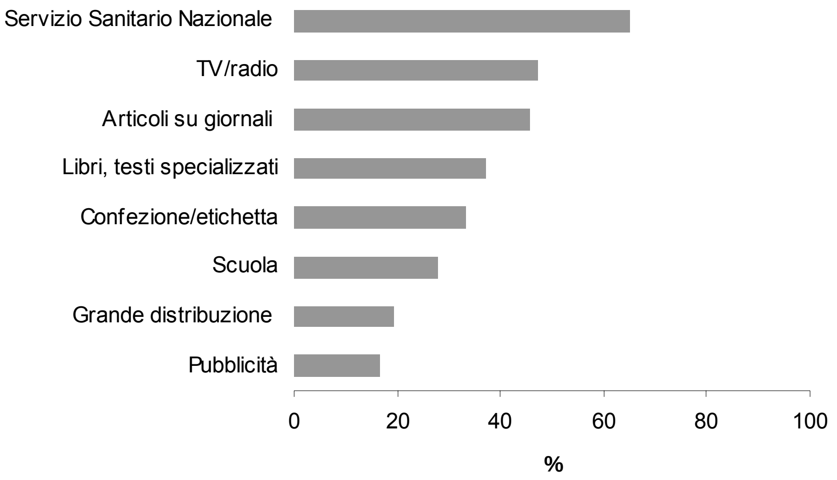

Cambiamenti apportati dopo la nascita del bambino

C'è una maggiore sensibilità dopo la nascita del figlio da parte dei genitori a cambiare abitudini alimentari: essi infatti hanno dichiarato di aver aumentato il consumo di frutta e verdura (47%), acqua (36%), pesce (29%), formaggi, latte, yogurt (26%), di aver diminuito il consumo di grassi/cibi grassi (48%) e sale (24%) e di aver eliminato cibi con additivi/conservanti (45%) .

Cambiamenti apportati dopo la nascita del bambino
(Sorveglianza nutrizionale anno 2005)

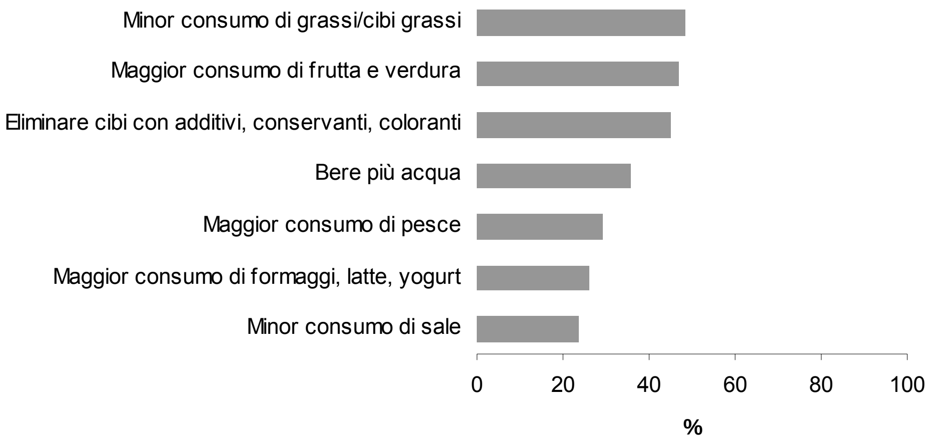

Fattori che influenzano le scelte alimentari

La scelta degli alimenti, come qualsiasi comportamento umano, è influenzata da molti fattori correlati. Gli impulsi fondamentali che ci spingono a mangiare o meno sono naturalmente la fame e la sazietà, ma ciò che scegliamo di mangiare non è determinato unicamente da bisogni fisiologici o nutrizionali. Altri fattori che influenzano la nostra scelta alimentare sono:

- le proprietà sensoriali dei cibi (gusto, odore o aspetto);
- fattori sociali, emozionali e cognitivi: preferenze e avversioni, conoscenze e atteggiamenti correlati alla dieta e alla salute, abitudine o contesto sociale. Per determinati individui, possono essere particolarmente importanti i valori personali, le esperienze di vita come il matrimonio/la convivenza, o le capacità (per es. culinarie), le convinzioni di una persona (per es. su temi come i cibi biologici e gli OGM), e le percezioni, quali le barriere percepite per seguire una dieta sana;
- fattori culturali, religiosi ed economici: l'istruzione, fattori etnici e la disponibilità, la visibilità o i prezzi dei prodotti rivestono un ruolo importante nelle nostre scelte.

Questa moltitudine di fattori illustra come "il mangiare sano", che è l'obiettivo delle campagne di salute pubblica, sia soltanto una delle molte considerazioni inerenti alla scelta del cibo.

La Pan-European Survey of Consumer Attitudes to Food, Nutrition and Health (Inchiesta paneuropea sugli atteggiamenti dei consumatori nei confronti di cibo, alimentazione e salute) ha rilevato che, in tutti gli stati membri europei, i cinque principali fattori che influenzano la scelta alimentare sono "qualità/freschezza" (74%), "prezzo" (43%), "gusto" (38%), "cercare di mangiare sano" (32%) e "quello che vuole mangiare la mia famiglia" (29%). Si tratta di risultati di media ottenuti raggruppando tutti i dati relativi agli stati membri europei, notevolmente diversi da Paese a Paese. Le donne, i soggetti più anziani e i più istruiti considerano particolarmente importanti gli

aspetti salutari. Per gli uomini “gusto” e “abitudine” sono con maggior frequenza i principali fattori che determinano la loro scelta alimentare. Il “prezzo” è l’elemento più importante per i disoccupati e i pensionati. Nella stessa inchiesta, l’80% degli intervistati ha descritto un’alimentazione sana (definita in base a “equilibrio” e “varietà”) in modo tale da far pensare che i messaggi sulla dieta abbiano un certo impatto. Questo si riflette in un certo miglioramento delle tendenze dietetiche. Tuttavia, la comprensione delle informazioni dietetiche non porta necessariamente all’azione. Ci deve essere la volontà effettiva di cambiare il comportamento personale. Ma l’esigenza percepita tra gli Europei di modificare le abitudini alimentari è ridotta, con il 71% che ritiene di avere una dieta adeguata. Questo conferma che la nutrizione/il mangiare sano non vengono percepiti come elementi di grande rilevanza nella scelta dei cibi.

Nello studio condotto, alla domanda su quali fattori influenzassero le scelte alimentari le risposte sono state: nel 72% dei casi “tentare di mangiare sano”, nel 58% “sapere cosa c’è dentro”, nel 51% la tendenza a soddisfare i gusti della famiglia o l’attenzione al gusto dei cibi e nel 40% la sicurezza alimentare.

Fattori che influenzano le scelte alimentari
(Sorveglianza nutrizionale anno 2005)

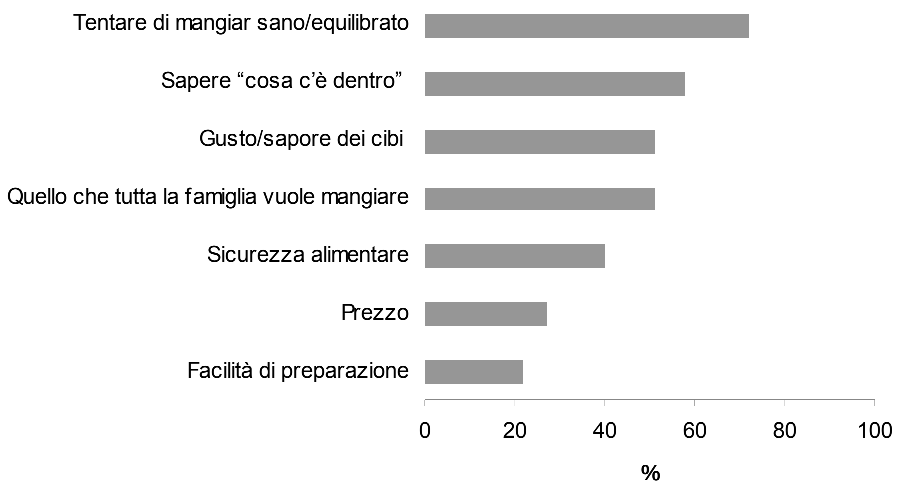

Maggiori difficoltà al mangiar sano

Una volta individuata la necessità del cambiamento, uno degli ostacoli all'adozione di un'alimentazione più sana è rappresentato dalle preferenze di gusto. La maggior parte delle persone considera i regimi alimentari sani monotoni, poco appetitosi o poco appaganti. La mancanza di tempo è citata frequentemente tra le cause che inducono a non seguire i consigli nutrizionali.

Negli ultimi anni, infatti, si assiste ad una sempre maggiore partecipazione della donna nel mercato del lavoro ed alla diffusione dell'orario di lavoro continuo o con pause ridotte per il pasto. Ciò porta ad una diversa organizzazione della famiglia e fa sì che il tempo diventi un fattore sempre più ridotto, inducendo le persone alla ricerca di prodotti alimentari di pronto consumo. E' in questa direzione che vanno cercate le ragioni alla base dell'aumento delle spese per prodotti surgelati, cibi semi-preparati, piatti pronti e non ultimo del ricorso al pasto fuori casa.

Un' educazione alimentare che dia indicazioni e suggerimenti su come sostituire con frutta e verdura gli alimenti abitualmente acquistati, senza che questo comporti un aumento del dispendio di tempo e denaro potrebbe rappresentare una soluzione percorribile. Anche gli sforzi di governi, autorità di salute pubblica, produttori e commercianti volti a promuovere piatti a base di frutta e verdura con un buon rapporto qualità/prezzo potrebbero dare un contributo positivo al cambiamento di dieta.

Anche nello studio condotto le difficoltà maggiormente dichiarate per effettuare una dieta più corretta sono state nel 53% dei casi i gusti della famiglia e nel 37% la vita molto impegnata e quindi poco tempo per preparare i pasti .

Difficoltà nell'adottare dieta più corretta
(Sorveglianza nutrizionale anno 2005)

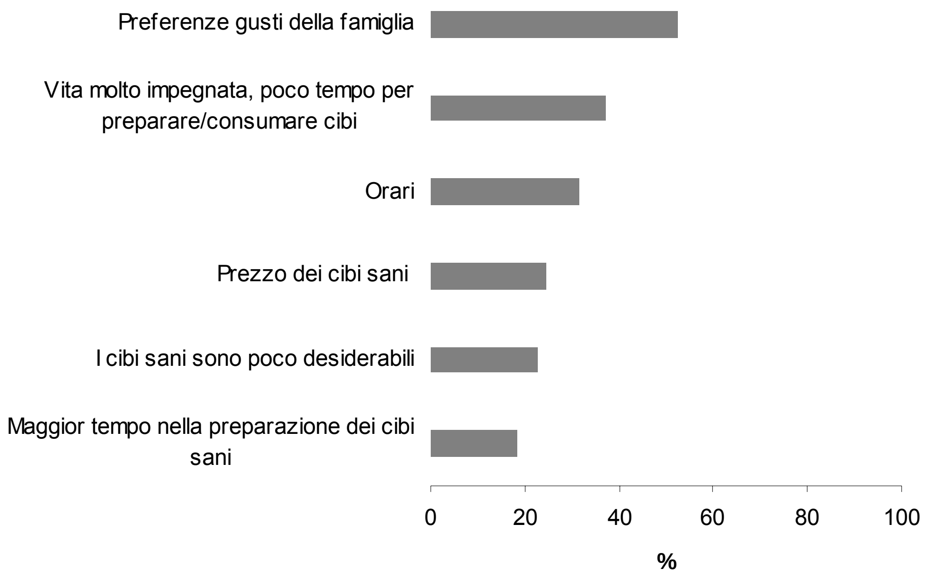

In particolare i maggiori ostacoli dichiarati al consumo degli alimenti considerati protettivi sono stati:

- per i legumi e le verdure il non gradimento ("non piacciono" nel 65% e nel 36% dei casi rispettivamente)
- per la frutta il fatto che non sempre è disponibile (31%)
- per il pesce il costo elevato (89%)

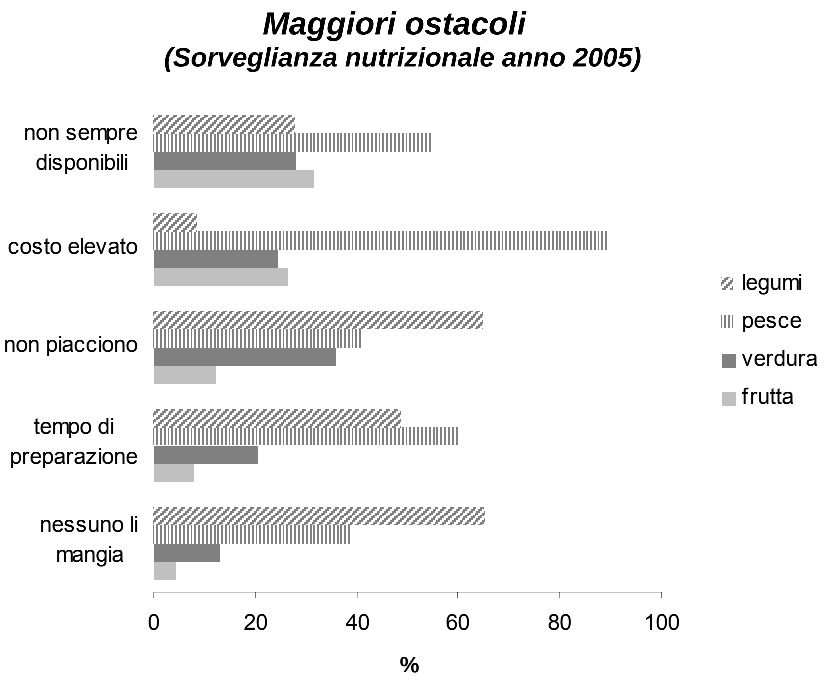

Esaminando la tipologia degli alimenti utilizzati, prevalgono sempre quelli freschi (99% dei casi), ma aumenta il consumo dei prodotti surgelati: dal 51% dei bambini di 6 anni al 56% dei bambini di 9 anni. La tendenza è contraria per quanto riguarda il consumo di prodotti biologici, che si riduce dal 26% al 18%.

I bambini e l’alimentazione

Le conoscenze

L’analisi dei questionari dei bambini è stata importante per capire il loro livello di conoscenze ed ha portato molti spunti interessanti per la progettazione di interventi futuri di educazione e promozione della salute.

Le informazioni che emergono da questo tipo di valutazione possono essere di aiuto nell’affrontare in maniera idonea atteggiamenti negativi e barriere potenziali che spesso si riscontrano nella realizzazione di interventi di educazione alimentare volti a determinare cambiamenti comportamentali.

E’ stata posta ai bambini la domanda *“Quanto sai sulla nutrizione?”* e, sebbene il 74% dei bambini abbia risposto “poco” (vs 62% a livello regionale) e il 6% “non so nulla” (vs 10% a livello regionale), in realtà hanno risposto in maniera molto competente alle domande successive, relative a come migliorare la loro alimentazione ed ancora di più alla loro percezione dell’effetto positivo o negativo degli alimenti sullo stato di salute.

Quanto sai sulla nutrizione?
(Sorveglianza nutrizionale anno 2005)

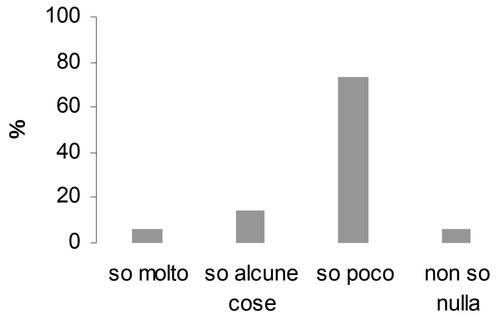

In particolare, alla domanda riguardante gli alimenti che dovrebbero mangiare in quantità maggiori, in più del 60% dei casi i bambini hanno risposto: pesce, frutta, minestre di verdura, frullati, legumi, verdure cotte. Alla domanda relativa ai cibi che dovrebbero mangiare di meno, in più del 40% dei casi la risposta è stata: cioccolato, dolciumi, bevande zuccherate, patatine, gelato e dolci.

Dovrei mangiare
(Sorveglianza nutrizionale anno 2005)

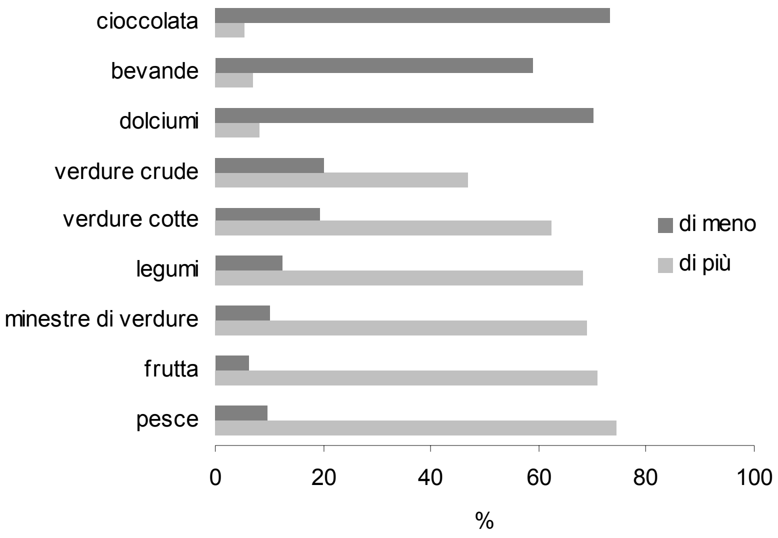

Alla domanda su come piacerebbe loro imparare la nutrizione, inoltre, hanno risposto mettendo ai primi posti i laboratori di cucina, le visite in fattoria e i programmi televisivi e agli ultimi i giornalini o i materiali informativi, privilegiando quindi il "fare" sul "conoscere" così come tutti i bambini della regione.

Queste informazioni ci potranno essere molto utili quando dovremmo predisporre interventi rivolti a bambini di questa classe di età.

Come ti piacerebbe imparare
(Sorveglianza nutrizionale anno 2005)

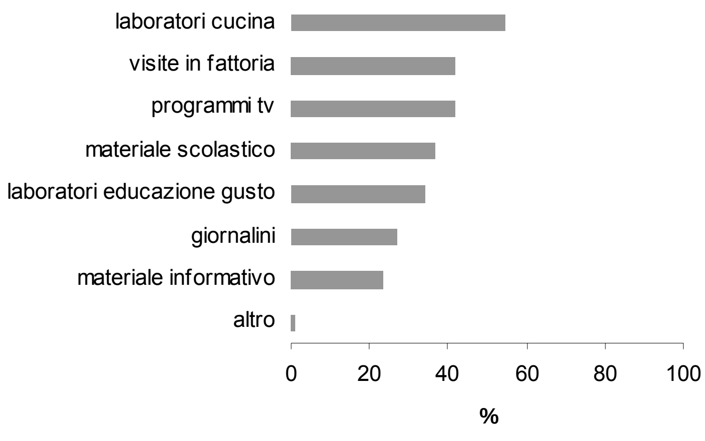

Per quanto riguarda il tema alimentare, i bambini hanno opinioni ben consolidate su alcune tematiche ormai oggetto di numerose campagne di informazione che riguardano i corretti principi nutrizionali.

Sei d'accordo?
(Sorveglianza nutrizionale anno 2005)

	No (%)	*Sì (%)*	*Non so (%)*
Per rimanere sano devi mangiare meno grassi	8	87	5
Il latte fa le ossa forti	1	85	13
La tua salute durante la crescita dipende dal cibo che mangi	3	85	13
E' importante mangiare cibi come pane e cereali integrali	5	81	13
Per crescere sano lo sport e l'attività motoria sono importanti come il cibo che mangi	8	81	11
Va bene mangiare cibi come caramelle e gelato, ma non tutti i giorni	19	76	6
E' meglio mangiare piccole quantità di cibi differenti anziché molto di un cibo solo	10	70	20
Frutta e verdura conservata sono nutrienti quanto frutta e verdura fresca	48	33	19
Le bibite gassate dissetano come l'acqua	85	7	8
Andare tutte le settimane al fast-food va bene	83	6	11
Va bene mangiare il cioccolato ogni giorno	95	4	1

Fa molto bene
(Sorveglianza nutrizionale anno 2005)

Alimenti	%
Frutta	93
Pesce	85
Minerali	78
Acqua	74
Carne	73
Legumi	73
Verdure crude	68
Latte	68
Verdure cotte	63
Formaggi	48

Non fa bene
(Sorveglianza nutrizionale anno 2005)

Alimenti	%
Dolci	97
Hamburger	87
Bibite	87
Cioccolato	85
Patate confezionate	83
Torte	71
Gelato	71
Merendine	67
Burro	60
Salumi	40

Giornata alimentare secondo i bambini

Dall'incrocio delle risposte date sui pasti consumati con quelle date sui pasti che loro ritenevano giusti, sono emerse queste considerazioni:

- per la colazione, il pranzo e la cena esiste una sostanziale correlazione tra quello che fanno e quello che ritengono giusto
- per lo spuntino della mattina e del pomeriggio un 25-30% di bambini non lo ritengono giusto anche se lo fanno, non riconoscendo l'importanza nutrizionale di interrompere il digiuno tra il pranzo e la cena
- per il dopocena o i fuori pasto c'è una consapevolezza della non correttezza di questi pasti e solo rispettivamente l'8% o il 6% lo ritengono giusto.

Pasti
(Sorveglianza nutrizionale anno 2005)

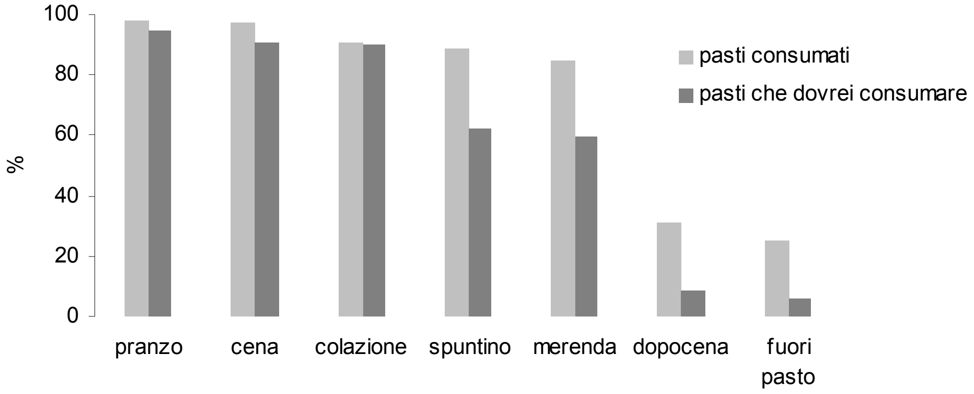

Alimenti graditi dai bambini

E' stato sondato sia il gradimento dei bambini per alcuni alimenti sia se siano mai stati assaggiati.

Il 33% dei bambini dichiara di non aver mai assaggiato i broccoli, il 27% la frutta esotica e il 22% i carciofi.

Fra le verdure il 78% dei bambini ha dichiarato di amare molto le carote, il 65% i finocchi, e il 58% pomodori e insalata, mentre non piacciono i broccoli (57%), il cavolfiore (52%), i peperoni (50%), e i carciofi (51%)

Tra la frutta piacciono molto pesche e albicocche (83%) e pere e mele (77%).

Tra i pesci piacciono molto il tonno (70% dei casi) e i bastoncini (69%) mentre non piace il polpo o la seppia (45%).

Mai assaggiato
(Sorveglianza nutrizionale anno 2005)

Alimenti	*%*
broccoli	33
frutta esotica	27
carciofi	22
cavolfiore	21
polpo/seppia	19
frutta secca	18
sogliola/platessa	18
sedano	17
filetto/trancio di pesce	15
peperoni	15

Mi piace molto
(Sorveglianza nutrizionale anno 2005)

Verdure	*%*
carote	78
finocchi	65
insalata e pomodori	58
Frutta	
albicocche/pesche	83
pera/mela	77
uva	75
Pesce	
tonno in scatola	71
bastoncini di pesce	69
vongole/cozze	64

Non mi piace per niente
(Sorveglianza nutrizionale anno 2005)

Verdure	*%*
broccoli	57
cavolfiore	52
carciofi	51
Frutta	
frutta secca	23
frutta esotica	18
melone	18
Pesce	
polpo/seppia	45
filetto/trancio di pesce	29
sogliola/platessa	29

Igiene orale

La carie dentale è la patologia dei denti più diffusa; è una malattia per cui gli acidi prodotti dai batteri dissolvono i denti con un processo di demineralizzazione: i batteri si servono dei carboidrati fermentabili (zuccheri e altri carboidrati contenuti negli alimenti e nelle bevande) per formare gli acidi che dissolvono i minerali come il calcio e il fosfato contenuti nei denti.

Al processo di remineralizzazione contribuisce la saliva, pulendo i residui di cibo dalla bocca, neutralizzando gli acidi prodotti dai batteri, fornendo calcio e fosfato ai denti e il fluoro inibendo la produzione di acido.

La carie dentaria si verifica soltanto quando il processo di demineralizzazione è più intenso di quello di remineralizzazione nell'arco di un certo periodo di tempo.

I seguenti fattori hanno conseguenze importanti per la salute dentale:

- Fattori individuali (forma della mandibola e della cavità orale, struttura dei denti, quantità e qualità della saliva)
- Igiene orale e uso di fluoro

  Si ritiene che la pulizia dei denti con dentifricio al fluoro sia il fattore più importante nel calo della carie dentaria osservato in molti Paesi. L'uso dello spazzolino e del filo interdentale, abbinato alla contemporanea applicazione di fluoro, aiuta ad eliminare i batteri dalla bocca riducendo il rischio di carie. I controlli dentistici regolari possono contribuire a rilevare e tenere sotto controllo i potenziali problemi. Il controllo e la rimozione regolari della placca batterica possono aiutare a diminuire l'incidenza della carie dentaria. Se la quantità di placca presente è molto scarsa, la quantità di acido che si forma è insignificante e la carie non può svilupparsi.
- Fattori alimentari

  Le abitudini alimentari, le caratteristiche degli alimenti e la frequenza del consumo continuano a influire sul rischio di insorgenza di questa malattia; i cibi che aderiscono ai denti presentano un rischio maggiore rispetto ai cibi che vengono eliminati rapidamente dalla bocca; le patatine e i biscotti per esempio, rimangono attaccati ai denti per un periodo più lungo rispetto alle caramelle e alle gelatine, forse per il fatto che queste ultime contengono zuccheri solubili che vengono lavati via dalla saliva più rapidamente. Più a lungo i cibi contenenti carboidrati rimangono sui denti, più tempo hanno i batteri per produrre acido e maggiori sono le probabilità di demineralizzazione. Anche se il legame con la frequenza di consumo sembra molto minore da quando è stata adottata una corretta igiene orale e si è diffusa l'assunzione di fluoro, ogni volta che mangiamo o beviamo qualcosa che contiene carboidrati i batteri cariogeni presenti sul dente incominciano a produrre acido, iniziando la demineralizzazione. Se si introducono cibi o bevande con troppa frequenza, lo smalto dei denti non ha la possibilità di remineralizzarsi completamente e la carie può incominciare a svilupparsi. Ecco perché è sconsigliabile continuare a mangiucchiare o sorseggiare per tutta la giornata.

Dalle risposte dei bambini di 9 anni emerge che il 55% dei bambini si lava i denti dopo i pasti principali, nel 92% dei casi viene usato il dentifricio mentre solo nel 18% il filo interdentale e solo il 19% dei bambini spazzola i denti per più di due minuti.

Quante volte lavi i denti?
(Sorveglianza nutrizionale anno 2005)

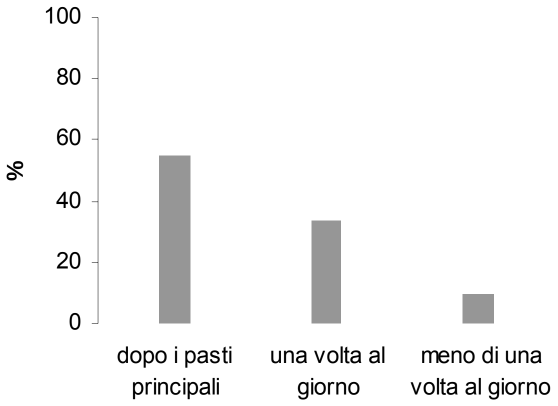

Cosa usi?
(Sorveglianza nutrizionale anno 2005)

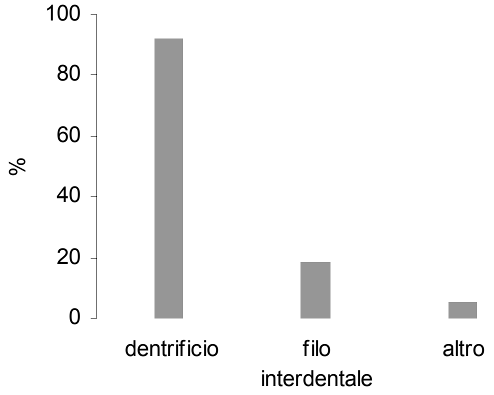

Conclusioni

Il sovrappeso e l'obesità nella popolazione infantile della regione Emilia-Romagna rappresentano un problema di grande rilevanza. Dalla ricerca risulta chiaro che sono molti i fattori coinvolti nella genesi dell'eccesso di peso in tale fascia di età: ambientali, socioculturali, cognitivi, comportamentali, relazionali, affettivi, etici tra loro in stretta relazione. Sono necessarie strategie diverse quindi per determinare un cambiamento nei comportamenti di gruppi con priorità diverse.

Viene confermata l'importanza primaria dell'attività motoria nella prevenzione dell'eccesso di peso nella popolazione infantile e ribadito quanto sia rischioso l'uso di televisione, computer e videogiochi per più di due ore al giorno.

E' stato infatti ampiamente dimostrato in letteratura una correlazione positiva tra il tempo passato a guardare la TV e lo sviluppo di obesità.

I meccanismi responsabili di questa associazione sono principalmente due: la tendenza alla sedentarietà e l'influenza della pubblicità verso un'alimentazione ipercalorica e squilibrata.

L'utilizzo invece di campagne d'informazione capaci di trasmettere valori positivi e socialmente utili, come quelle relative all'educazione alimentare, troverebbero un terreno fertile in quanto nei bambini troviamo fattori quali la facile permeabilità nei confronti dei messaggi, l'attenzione rivolta a captare informazioni per affrontare il mondo e la capacità crescente d'influenzare le scelte familiari.

Dagli stessi bambini sono emersi però anche la necessità ed il bisogno di sentirsi coinvolti in modo attivo, sia in famiglia che a scuola, nelle iniziative per acquisire nuove informazioni.

In generale si è evidenziata l'importanza di intervenire più sui comportamenti che sulle conoscenze, sia per quanto riguarda i bambini che gli adulti.

Buone abitudini, comportamenti corretti e stili di vita adeguati, se acquisiti sin da bambini, costituiscono la base per garantire la salute. L'efficacia di interventi educativi è quindi strettamente legata al coinvolgimento precoce e attivo prima dei genitori e poi della scuola, sede elettiva di interventi che mirano al raggiungimento di stili di vita salutari. Occorre però che oltre alla famiglia ed alla scuola le istituzioni tutte, il mondo produttivo e tutte le componenti che gravitano attorno al bambino, lavorino insieme in modo sinergico, integrato, con obiettivi condivisi e coerenti per promuovere benessere. Si dovranno stringere alleanze tra governi, autorità locali e autorità di salute pubblica per creare un ambiente che favorisca il cambiamento dello stile di vita e integri i messaggi sull'alimentazione e i programmi di educazione.

Bibliografia

1. Ben…essere in tavola principi nutrizionali e alimenti: istruzioni per l'uso a scuola e a casa Gruppo interistituzionale provinciale per l'educazione alimentare della provincia di Forlì-Cesena http://www.provincia.forli-cesena.it/agricoltura/frame.htm
2. Cole TJ, Bellizzi MC, Flegal KM, Dietz WH (2000): Establishing a standard definition for child overweight and obesity worldwide: international survey, BMJ 320, (7244), 1240-3.
3. Conferenza Ministeriale della Regione Europea dell'OMS sull'azione di Contrasto all'Obesità: Carta Europea sull'azione di contrasto all'obesità Istambul, Turchia, 15-17 novembre 2006
4. Diet, Nutrition and the Prevention of Chronic Diseases. WHO, Ginevra 2003.
5. Dossier dell'Azienda USL di Cesena: Sorveglianza nutrizionale nelle scuole materne dell'Azienda Sanitaria di Cesena - Novembre 2004.
6. Dossier dell'Azienda USL di Ferrara: Sorveglianza nutrizionale nelle scuole materne ed elementari dell'Azienda Unità Sanitaria Locale di Ferrara - Novembre 2006.
7. Ferraroni M (1993): Possibilità di utilizzo di questionari alimentari abbreviati nelle indagini epidemiologiche. Giornale Europeo di Nutrizione clinica. 2, (2), 39-40.
8. Ferro-Luzzi A & Leclercq C (1993): Nutritional surveillance: an outline. In: Food and Nutrition Policy in Europe, eds. Van der Heij DG, Lowik MRH, Ochuisen T, 113-117. The Hague Wageningen: Pudoc Scientific.
9. Gruppo Regionale Nutrizione Regione Emilia-Romagna: Il rischio di obesità infantile nella Regione Emilia-Romagna Pediatria Preventiva e Sociale supplemento numero 1/2006 5-12
10. http://www.eufic.org/page/it/malattie-legate-alimentazione/igiene-dentale/
11. http://www.eufic.org/page/it/malattie-legate-alimentazione/obesita/
12. http://www.eufic.org/page/it/salute-e-stile-di-vita/attivita-fisica/
13. http://www.ministerosalute.it/resources/static/pubblicazioni/obiettivi_salute.pdf
14. http://www.sanita.toscana.it/promozione/report_hbsc.shtml
15. Il Piano regionale della prevenzione dell'Emilia-Romagna Intesa Stato-Regioni e Province Autonome 23 marzo 2005 Servizio Sanitario Regionale Emilia-Romagna Collana Contributi 44
16. International Obesity Taskforce. www.iotf.org
17. ISTAT Indagine Multiscopo sulle famiglie Aspetti della vita quotidiana "Stili di vita e condizioni di salute" ANNO 2003.
18. ISTAT Indagine Multiscopo sulle famiglie"Condizioni di salute e ricorso ai servizi sanitari" Anni 1999-2000.
19. Iughetti L, Bernasconi S: L'obesità in età evolutiva. Ed. McGraw-Hill - 2005.
20. Leclerq C, Martino L & Ferro-Luzzi A (1995): La sorveglianza nutrizionale: come e perché. Giornale italiano di Chimica Clinica. 20, (2), 87-97.
21. Linee Guida per una sana alimentazione INRAN Edizione 2003.
22. Manuale di Sorveglianza Nutrizionale INRAN 2003.
23. Obesità infantile. Ministero della Salute; www.ministerosalute.it, 2003.
24. Società Italiana di Nutrizione Umana: Prevenire l'Obesità in Italia. Ed. EDRA Medical Publishing & New Media - 2001.

25. Sorveglianza nutrizionale Determinanti e indicatori di rischio obesità nella popolazione infantile Emilia-Romagna Indagine 2003 e 2005 Servizio Sanitario Regionale Emilia-Romagna Collana Contributi 49

26. WHO: Global strategy on diet, physical activity and health. Fifty-seventh world health assembly - 2004.

27. World Health Organization (1985): Use and interpretation of anthropometric indicators of nutritional status. WHO Working Group. Bull World health organ. 64, (6), 929-41.

28. World Health Organization Study Group (1976): Methodology of nutritional surveillance. Report of a joint FAO/UNICEF/WHO Expert Committee. World Health Organ Tech Rep Ser. 593, 1-66.

Allegato n°1

Questionario somministrato ai genitori dei bambini di 6 anni

Allegato n°1

Questionario somministrato ai genitori dei bambini di 6 anni

AZIENDA U. S. L. di CESENA Regione Emilia Romagna

Studio So.N.I.A. Sorveglianza Nutrizionale Infanzia Adolescenza ☐☐☐☐☐☐☐☐

Rileviamo questi dati anonimi per motivi statistici ed epidemiologici nel pieno rispetto della legge 675/96 sulla riservatezza. Per ciascuna domanda La preghiamo di leggere sempre fino in fondo tutte le risposte. La ringraziamo per la collaborazione.

Scuola ______________________
Comune ______________________ PROV ______

Spazio riservato al rilevatore
Data di compilazione ___/___/___
Peso ________ **Kg** **Altezza** ________ **cm**

Il Bambino

**Legga le seguenti affermazioni e per ciascuna scelga:**	**Vero**	**Falso**
Mio figlio mangia molto soprattutto cibi ricchi e appetitosi	☐	☐
Mi risulta molto difficile variare l'alimentazione di mio figlio	☐	☐
A mio figlio piace fare tanti piccoli spuntini nell'arco della giornata	☐	☐
Mio figlio ama un'alimentazione molto varia	☐	☐
Far mangiare mio figlio è sempre un'impresa molto difficile	☐	☐
Mio figlio bada più alla quantità che alla qualità del cibo	☐	☐
Il menù di mio figlio è monotono	☐	☐
Mio figlio mangia soprattutto fuori pasto	☐	☐
Mio figlio mangia volentieri tutto ciò che gli do	☐	☐
Devo usare molta fantasia per stimolare mio figlio a mangiare	☐	☐

Quando Suo figlio rifiuta un cibo come si comporta?
☐ Lo obbliga a mangiare
☐ Propone un altro cibo
☐ Cerca di far accettare i cibi sgraditi
☐ Non insiste

Suo figlio Le chiede di comperare cibi che vede pubblicizzati in TV?
☐ Sempre ☐ Spesso ☐ Qualche volta ☐ Mai

... e lei lo accontenta?
☐ Sempre ☐ Spesso ☐ Qualche volta ☐ Mai

Data di nascita ____/____/____
Maschio☐ **Femmina** ☐

Peso alla nascita del bambino: (Kg) ……………….
Allattamento :
☐ materno ☐ artificiale ☐ misto

Suo figlio ha problemi di allergie o intolleranze alimentari? ☐ No ☐ Si, indicare quali
☐ Cutanei (orticaria,…)…………………
☐ Respiratori (riniti,…)…………………
☐ Gastrointestinali ……………………
☐ Altro (specificare) ……………………

Suo figlio segue restrizioni/scelte dietetiche? ☐ No
Si, indicare quali
☐ Motivi Religiosi……………………………
☐ Motivi Etici…………………………………
☐Motivi Sanitari(specificare)……………………

Suo figlio fa uso di integratori alimentari?

	No	Saltuariamente	Spesso
Vitamine	☐	☐	☐
Minerali	☐	☐	☐
Altro (Propoli, Ginseng…)	☐	☐	☐

Specificare………………………………………

La Famiglia

Provenienza geografica della Madre
Regione italiana (specificare)……………………………
Stato estero (specificare)……………………………

Provenienza geografica del Padre
Regione italiana (specificare)……………………………
Stato estero (specificare)……………………………

Grado di istruzione conseguito

Madre	Padre	
☐	☐	Nessuno
☐	☐	Licenza elementare
☐	☐	Licenza media
☐	☐	Diploma
☐	☐	Laurea

Professione

Madre	Padre	
☐	☐	Imprenditore, dirigente, libero professionista
☐	☐	Impiegato, quadro, tecnico, insegnante
☐	☐	Commerciante, esercente, artigiano
☐	☐	Operaio, commesso, bracciante
☐	☐	Casalinga/o, pensionata/o
☐	☐	In cerca di occupazione
☐	☐	Altro (Specificare)…………………

Età

Madre	Padre	
☐	☐	Meno di 30
☐	☐	Tra 30 e 39
☐	☐	Tra 40 e 49
☐	☐	50 ed oltre

Vivono in famiglia
☐ Nonno ☐ Nonna
☐ Altro: (specificare)………………
Numero figli totale |_|_|
Totale componenti: |_|_|

Dati antropometrici
Madre Altezza ________ cm Peso ________ kg
Padre Altezza ________ cm Peso ________ kg

Le Attività di una Giornata "Tipo"

Descriva una giornata standard (24 ore)di ***suo figlio*** *suddividendola nelle diverse attività:*

SONNO	_____ore/giorno+
SCUOLA	_____ore/giorno +
ATTIVITA' LEGGERA (Tv, leggere, mangiare, lavarsi, treno/bus, videogiochi, giochi tranquilli…)	_____ore/giorno+
ATTIVITA' VIVACE (Camminare, bicicletta, ginnastica, danza, giochi attivi …)	_____ore/giorno+
**Può per favore verificare il TOTALE:**	**24 ore/giorno**

Pratica attività motoria/sportiva organizzata? NO ☐ SI ☐ *Se SI può indicare quale e stimarne le ore **settimanali**?*

Attività motoria/sportiva praticata (specificare)________________________ N° ore a settimana_____

La Giornata Alimentare del Bambino

LA PRIMA COLAZIONE

Quante volte suo figlio consuma la PRIMA COLAZIONE durante la settimana?
☐ Tutti i giorni (7 volte) ☐ Quasi tutti i giorni (4-6 volte) ☐ A volte (1-3 volte) ☐ Mai

Di solito con quali **ALIMENTI** ? *(risposte multiple)*
- ☐ Frutta
- ☐ Pane, fette biscottate, …
- ☐ Prodotti da forno dolci (biscotti, merendine, brioches, torte, …)
- ☐ Prodotti da forno salati (crackers, pizza, crescente, …)
- ☐ Fiocchi di cereali
- ☐ Miele o marmellata, crema spalmabile
- ☐ Uova, formaggi, salumi
- ☐ Altro (specificare)……………………………………………………

e **BEVANDE** ? *(risposte multiple)*
- ☐ Latte/latte aromatizzato (con cacao,…)
- ☐ Thè
- ☐ Yogurt
- ☐ Succo di frutta o spremuta
- ☐ Acqua
- ☐ Altro ……………………………………………
……………………………………………………
……………………………………………………

SPUNTINO DELLA MATTINA

Quante volte suo figlio consuma lo SPUNTINO durante la settimana?
☐ Tutti i giorni (7 volte) ☐ Quasi tutti i giorni (4-6 volte) ☐ A volte (1-3 volte) ☐ Mai

Di solito con quali **ALIMENTI** ? *(risposte multiple)*
- ☐ Frutta
- ☐ Prodotto da forno dolce (biscotti, merendine, brioches, torte…)
- ☐ Prodotto da forno salato (crackers, pizza, crescente, …)
- ☐ Panino imbottito (con formaggi, salumi, creme spalmabili dolci/salate…)
- ☐ Snack dolce (cioccolato, barrette,…)
- ☐ Snack salato (patatine, pop corn, salatini, soffiati al formaggio …)
- ☐ Altro (specificare)…………………………………………………………

e **BEVANDE** ? *(risposte multiple)*
- ☐ Latte/latte aromatizzato (con cacao,…)
- ☐ Yogurt
- ☐ Succo di frutta o spremuta
- ☐ Bibite gassate
- ☐ Thè o tisana
- ☐ Acqua
- ☐ Altro ……………………………………………

IL PRANZO

Di solito è costituito da : *(risposte multiple)*
- ☐ Primo
- ☐ Secondo
- ☐ Contorno
- ☐ Frutta
- ☐ Dolce
- ☐ Altro ………………………………………………………………

e da quali **BEVANDE** ?*(risposte multiple)*
- ☐ Latte/latte aromatizzato (con cacao,…)
- ☐ Succo di frutta o spremuta
- ☐ Bibite gassate
- ☐ Thè o tisana
- ☐ Acqua
- ☐ Altro ……………………………………………

SPUNTINO POMERIDIANO

Quante volte suo figlio consuma lo SPUNTINO durante la settimana?
☐ Tutti i giorni (7 volte) ☐ Quasi tutti i giorni (4-6 volte) ☐ A volte (1-3 volte) ☐ Mai

Di solito con quali **ALIMENTI** ? *(risposte multiple)*
- ☐ Frutta
- ☐ Prodotto da forno dolce (biscotti, merendine, brioches, torte, …)
- ☐ Prodotto da forno salato (cracker, pizza, crescente, …)
- ☐ Panino imbottito (con formaggi, salumi, creme spalmabili dolci, salate…)
- ☐ Snack dolce (cioccolato, barrette, gelati, torrone…)
- ☐ Snack salato (patatine, pop corn, salatini, soffiati al formaggio …)
- ☐ Altro ……………………………………………………………

e da quali **BEVANDE** ? *(risposte multiple)*
- ☐ Latte/latte aromatizzato (con cacao,…)
- ☐ Yogurt
- ☐ Succo di frutta o spremuta
- ☐ Bibite gassate
- ☐ Thè o tisana
- ☐ Acqua
- ☐ Altro ……………………………………………

LA CENA

Di solito è costituita da : *(risposte multiple)*
- ☐ Primo
- ☐ Secondo
- ☐ Contorno
- ☐ Frutta
- ☐ Dolce
- ☐ Altro ……………………………………………………………………
……………………………………………………………………………

e da quali **BEVANDE** ? *(risposte multiple)*
- ☐ Latte/latte aromatizzato (con cacao,…)
- ☐ Yogurt
- ☐ Succo di frutta o spremuta
- ☐ Bibite gassate
- ☐ Thè o tisana
- ☐ Acqua
- ☐ Altro ……………………………………………

DOPO LA CENA

Suo figlio mangia e beve dopo la cena? ☐ Tutti i giorni (7 volte) ☐ Quasi tutti i giorni (4-6) volte ☐ A volte (1-3 volte) ☐ Mai

Di solito quali **ALIMENTI** consuma? *(risposte multiple)*
- ☐ Frutta
- ☐ Prodotto da forno dolce (biscotti, merendine, brioches, torte,, …)
- ☐ Prodotto da forno salato (crackers, pizza, crescente, …)
- ☐ Panino imbottito (con formaggi, salumi, creme spalmabili dolci, salate…)
- ☐ Snack dolce (cioccolato, barrette, gelati, torrone…)
- ☐ Snack salato (patatine, pop corn, salatini, soffiati al formaggio…)
- ☐ Altro (specificare)……………………………………………………
……………………………………………………………………

e quali **BEVANDE** ? *(risposte multiple)*
- ☐ Latte/latte aromatizzato (con cacao,…)
- ☐ Yogurt
- ☐ Succo di frutta o spremuta
- ☐ Bibite gassate
- ☐ Thè, Tisane, camomilla..
- ☐ Acqua
- ☐ Altro (specificare) ……………………………

Frequenze di Consumo

CEREALI&TUBERI	Mai	Meno di 1 volta al mese	1-3 volte mese	1 volta a sett.	2-4 volta a sett.	5-6 volte a sett.	1 volta a giorno	2-3 volte al giorno	4-5 volte al giorno
Pasta	☐	☐	☐	☐	☐	☐	☐	☐	☐
Pasta ripiena	☐	☐	☐	☐	☐	☐	☐	☐	☐
Riso, orzo	☐	☐	☐	☐	☐	☐	☐	☐	☐
Pane	☐	☐	☐	☐	☐	☐	☐	☐	☐
Grissini/crackers	☐	☐	☐	☐	☐	☐	☐	☐	☐
Pizza/focaccia	☐	☐	☐	☐	☐	☐	☐	☐	☐
Patatine, pop corn salatini	☐	☐	☐	☐	☐	☐	☐	☐	☐
Patate	☐	☐	☐	☐	☐	☐	☐	☐	☐
Altro........................	☐	☐	☐	☐	☐	☐	☐	☐	☐
**DOLCI&DOLCIUMI**									
Gelato	☐	☐	☐	☐	☐	☐	☐	☐	☐
Biscotti	☐	☐	☐	☐	☐	☐	☐	☐	☐
Merendine	☐	☐	☐	☐	☐	☐	☐	☐	☐
Torte/paste	☐	☐	☐	☐	☐	☐	☐	☐	☐
Zucchero (aggiunto)	☐	☐	☐	☐	☐	☐	☐	☐	☐
"Nutella", creme spalmabili	☐	☐	☐	☐	☐	☐	☐	☐	☐
Miele/marmellata	☐	☐	☐	☐	☐	☐	☐	☐	☐
Caramelle/gomme	☐	☐	☐	☐	☐	☐	☐	☐	☐
Barrette/Cioccolata	☐	☐	☐	☐	☐	☐	☐	☐	☐
**CARNI etc**	**Mai**	**Meno di 1 volta al mese**	**1-3 volte mese**	**1 volta a sett.**	**2-4 volta a sett.**	**5-6 volte a sett.**	**1 volta a giorno**	**2-3 volte al giorno**	**4-5 volte al giorno**
Bovine	☐	☐	☐	☐	☐	☐	☐	☐	☐
Suine	☐	☐	☐	☐	☐	☐	☐	☐	☐
Bianche (pollo, coniglio..)	☐	☐	☐	☐	☐	☐	☐	☐	☐
Ovine (agnello....)	☐	☐	☐	☐	☐	☐	☐	☐	☐
Equine, Struzzo	☐	☐	☐	☐	☐	☐	☐	☐	☐
Salumi	☐	☐	☐	☐	☐	☐	☐	☐	☐
Uova	☐	☐	☐	☐	☐	☐	☐	☐	☐
**PESCE**									
Mitili e Molluschi	☐	☐	☐	☐	☐	☐	☐	☐	☐
Crostacei (gamberi)	☐	☐	☐	☐	☐	☐	☐	☐	☐
Pesci con spina dorsale	☐	☐	☐	☐	☐	☐	☐	☐	☐
Pesce in scatola (tonno, ...)	☐	☐	☐	☐	☐	☐	☐	☐	☐
Bastoncini	☐	☐	☐	☐	☐	☐	☐	☐	☐
**LATTICINI**	**Mai**	**Meno di 1 volta al mese**	**1-3 volte mese**	**1 volta a sett.**	**2-4 volta a sett.**	**5-6 volte a sett.**	**1 volta a giorno**	**2-3 volte al giorno**	**4-5 volte al giorno**
Latte	☐	☐	☐	☐	☐	☐	☐	☐	☐
Yogurt	☐	☐	☐	☐	☐	☐	☐	☐	☐
Formaggio tipo "Grana"	☐	☐	☐	☐	☐	☐	☐	☐	☐
Formaggi freschi	☐	☐	☐	☐	☐	☐	☐	☐	☐
Formaggi stagionati	☐	☐	☐	☐	☐	☐	☐	☐	☐
**LEGUMI**									
Fagioli	☐	☐	☐	☐	☐	☐	☐	☐	☐
Lenticchie	☐	☐	☐	☐	☐	☐	☐	☐	☐
Ceci/Fave	☐	☐	☐	☐	☐	☐	☐	☐	☐
Piselli	☐	☐	☐	☐	☐	☐	☐	☐	☐
Soia	☐	☐	☐	☐	☐	☐	☐	☐	☐
**VERDURE**	**Mai**	**Meno di 1 volta al mese**	**1-3 volte mese**	**1 volta a sett.**	**2-4 volta a sett.**	**5-6 volte a sett.**	**1 volta a giorno**	**2-3 volte al giorno**	**4-5 volte al giorno**
Verdure in minestrone	☐	☐	☐	☐	☐	☐	☐	☐	☐
Pomodori/ in salsa	☐	☐	☐	☐	☐	☐	☐	☐	☐
Biete/spinaci	☐	☐	☐	☐	☐	☐	☐	☐	☐
Lattuga/insalate	☐	☐	☐	☐	☐	☐	☐	☐	☐
Carote	☐	☐	☐	☐	☐	☐	☐	☐	☐
Peperoni	☐	☐	☐	☐	☐	☐	☐	☐	☐
Carciofi/asparagi	☐	☐	☐	☐	☐	☐	☐	☐	☐
Cipolle/aglio	☐	☐	☐	☐	☐	☐	☐	☐	☐
Finocchi	☐	☐	☐	☐	☐	☐	☐	☐	☐
Zucchine/melanzane	☐	☐	☐	☐	☐	☐	☐	☐	☐
Fagiolini	☐	☐	☐	☐	☐	☐	☐	☐	☐
Cavolo/cavolfiore	☐	☐	☐	☐	☐	☐	☐	☐	☐
**FRUTTA**	**Mai**	**Meno di 1/mese**	**1-3 mese**	**1 a sett.**	**2-4 a sett.**	**5-6 a sett.**	**1 al giorno**	**2-3 al giorno**	**4-5 al giorno**
Agrumi/kiwi	☐	☐	☐	☐	☐	☐	☐	☐	☐
Mele/pere	☐	☐	☐	☐	☐	☐	☐	☐	☐
Banane	☐	☐	☐	☐	☐	☐	☐	☐	☐
Frutta estiva	☐	☐	☐	☐	☐	☐	☐	☐	☐
Frutta esotica	☐	☐	☐	☐	☐	☐	☐	☐	☐
Frutta di bosco	☐	☐	☐	☐	☐	☐	☐	☐	☐
Frutta secca	☐	☐	☐	☐	☐	☐	☐	☐	☐
**BEVANDE**									
Bibite gassate	☐	☐	☐	☐	☐	☐	☐	☐	☐
Spremute/succhi	☐	☐	☐	☐	☐	☐	☐	☐	☐
Tisane, Thè	☐	☐	☐	☐	☐	☐	☐	☐	☐

AL BIMBO PIACE ?

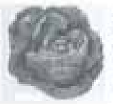

Molto
Abbastanza
Così così
Per niente

La lattuga

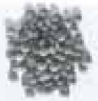

I legumi

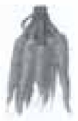

Le carote

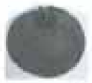

I pomodori

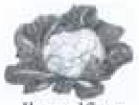

Il cavolfiore

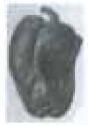

I peperoni

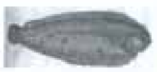

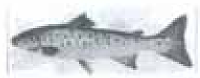

La trota

Allegato n°2

Questionario somministrato ai genitori dei bambini di 9 anni

Allegato n°2

Questionario somministrato ai genitori dei bambini di 9 anni

SERVIZIO SANITARIO REGIONALE
EMILIA-ROMAGNA

Regione Emilia Romagna

☐☐☐☐ **Studio So.N.I.A. Sorveglianza Nutrizionale Infanzia Adolescenza** ☐☐☐☐

*Rileviamo questi dati anonimi per motivi statistici ed epidemiologici nel pieno rispetto del **D.lgs.n.196/03** sulla riservatezza.*
Per ciascuna domanda La preghiamo di leggere sempre fino in fondo tutte le risposte. La ringraziamo per la collaborazione.

Scuola________________________ Comune________________ PROV____

Il bambino

**Legga le seguenti affermazioni e per ciascuna scelga se è VERO o FALSO**	**VERO**	**FALSO**
Mio figlio mangia molto soprattutto cibi ricchi e appetitosi	☐	☐
Mi risulta molto difficile variare l'alimentazione di mio figlio	☐	☐
Far mangiare mio figlio è sempre un'impresa molto difficile	☐	☐
Mio figlio mangia soprattutto fuori pasto	☐	☐
Mio figlio mangia volentieri tutto ciò che gli do	☐	☐
Devo usare molta fantasia per stimolare mio figlio a mangiare	☐	☐

Quando Suo figlio rifiuta un cibo come si comporta?
☐ Lo obbliga a mangiare
☐ Propone un altro cibo
☐ Cerca di far accettare i cibi sgraditi
☐ Non insiste

Quante ore al giorno suo figlio passa davanti alla TV ? _____ore/die

Suo figlio Le chiede di comperare cibi che vede pubblicizzati in TV?
☐ Sempre ☐ Spesso ☐ Qualche volta ☐ Mai

.... **e lei lo accontenta?**
☐ Sempre ☐ Spesso ☐ Qualche volta ☐ Mai

Peso alla nascita del bambino: (Kg) ....

Nato a ☐Termine ☐ Pretermine

Allattamento esclusivo al seno ☐No ☐ Si fino al …mese

Suo figlio ha problemi di allergie alimentari testate clinicamente? ☐ No ☐ Si

Suo figlio presenta intolleranze alimentari? ☐ No ☐ Si

Suo figlio segue restrizioni/scelte dietetiche? ☐ No
Si , indicare quali
☐ Motivi Religiosi.................................
☐ Motivi Etici (es.vegetariani)..................
☐ Motivi Sanitari(specificare)...................

**Suo figlio fa uso di....?**	No	Saltuariamente	Spesso	Sempre
Vitamine e Minerali	☐	☐	☐	☐
Fluoro in compresse	☐	☐	☐	☐
Dentifricio al fluoro	☐	☐	☐	☐

La famiglia

Provenienza geografica della Madre
Regione italiana (specificare)..
Stato estero (specificare)..

Provenienza geografica del Padre
Regione italiana (specificare)..
Stato estero (specificare)..

Grado di istruzione conseguito

Madre	Padre	
☐	☐	Nessuno
☐	☐	Licenza elementare
☐	☐	Licenza media
☐	☐	Diploma
☐	☐	Laurea

Professione

Madre	Padre	
☐	☐	Imprenditore, dirigente, libero professionista
☐	☐	Impiegato, quadro, tecnico, insegnante
☐	☐	Commerciante, esercente, artigiano
☐	☐	Operaio, commesso, bracciante
☐	☐	Casalinga/o, pensionata/o
☐	☐	In cerca di occupazione
☐	☐	Altro (Specificare)..................................

Età

Madre		Padre
☐	Meno di 30	☐
☐	Tra 30 e 39	☐
☐	Tra 40 e 49	☐
☐	50 ed oltre	☐

Vivono in famiglia
☐ Nonno ☐ Nonna
☐ Altro: (specificare).....................

Dati antropometrici ***(misurazioni recenti)***
Madre Altezza__________cm Peso______________kg
Padre Altezza__________cm Peso______________kg

Numero figli totale |_|_|
Totale componenti: |_|_|

La giornata "Tipo"

Descriva una giornata standard (24 ore)di ***suo figlio*** *suddividendola nelle diverse attività:*

ATTIVITA' VIVACE (Camminare, bicicletta, ginnastica, danza, giochi attivi ....) .... ore al giorno +
Ore di **ATTIVITA' LEGGERA** (Tv, leggere, mangiare, treno/bus, videogiochi, computer, giochi tranquilli....) .... ore al giorno +
Ore di **SCUOLA** ....ore al giorno +
Ore di **SONNO** ....ore al giorno =
Può per favore verificare il TOTALE: 24 ore al giorno

Sport e attività motoria

Suo figlio pratica attività motoria/sportiva organizzata?
NO ☐ *Per quale motivo?*..
....
....
....

SI ☐ *Può indicare quale e stimarne le ore* ***settimanali****?*

Attività motoria/sportiva praticata	N° ore a settimana
A..	A............
B..	B ....
C..	C............

La Giornata Alimentare del Bambino

LA PRIMA COLAZIONE

Quante volte suo figlio consuma la PRIMA COLAZIONE durante la settimana?
☐ Tutti i giorni (7 volte) ☐ Quasi tutti i giorni (4-6 volte) ☐ A volte (1-3 volte) ☐ Mai

Di solito con quali **ALIMENTI e BEVANDE**? *(risposte multiple)*

☐ Frutta, spremuta, centrifugato di frutta
☐ Pane, fette biscottate, …
☐ Prodotti da forno dolci (biscotti, merendine, brioches, torte, …)
☐ Prodotti da forno salati (crackers, pizza, crescente, …)
☐ Fiocchi di cereali
☐ Miele o marmellata, crema spalmabile
☐ Uova, formaggi, salumi
☐ Thè o tisana
☐ Yogurt
☐ Latte/latte aromatizzato (con cacao,…)
☐ Succo di Frutta
☐ Acqua
☐ Altro ……………………………………

SPUNTINO DELLA MATTINA

Quante volte suo figlio consuma lo SPUNTINO durante la settimana? ☐ Tutti i giorni (7 volte) ☐ Quasi tutti i giorni (4-6 volte) ☐ A volte (1-3 volte) ☐ Mai

Di solito con quali **ALIMENTI e BEVANDE** ? *(risposte multiple)*

☐ Frutta, spremuta, centrifugato di frutta
☐ Prodotto da forno dolce (biscotti, merendine, brioches, torte…)
☐ Prodotto da forno salato (crackers, pizza, crescente, …)
☐ Panino imbottito (con formaggi, salumi, creme spalmabili dolci/salate…)
☐ Snack dolce (cioccolato, barrette,…)
☐ Snack salato (patatine, pop corn, salatini, soffiati al formaggio …)
☐ Latte/latte aromatizzato (con cacao,…)
☐ Yogurt
☐ Succo di frutta
☐ Bibite gassate
☐ Thè o tisana
☐ Acqua
☐ Altro ……………………………………

IL PRANZO

Di solito è costituito da : *(risposte multiple)*

☐ Primo
☐ Secondo
☐ Contorno
☐ Frutta, spremuta o centrifugato di frutta
☐ Dolce
☐ Acqua
☐ Succo di frutta
☐ Bibite gassate
☐ Thè o tisana
☐ Latte/latte aromatizzato (con cacao,…)
☐ Altro ……………………………………

SPUNTINO POMERIDIANO

Quante volte suo figlio consuma lo SPUNTINO durante la settimana? ☐ Tutti i giorni (7 volte) ☐ Quasi tutti i giorni (4-6 volte) ☐ A volte (1-3 volte) ☐ Mai

Di solito con quali **ALIMENTI e BEVANDE** ? *(risposte multiple)*

☐ Frutta, spremuta, centrifugato di frutta
☐ Prodotto da forno dolce (biscotti, merendine, brioches, torte, …)
☐ Prodotto da forno salato (cracker, pizza, crescente, …)
☐ Panino imbottito (con formaggi, salumi, creme spalmabili dolci, salate…)
☐ Snack dolce (cioccolato, barrette, gelati, torrone…)
☐ Snack salato (patatine, pop corn, salatini, soffiati al formaggio …)
☐ Latte/latte aromatizzato (con cacao,…)
☐ Yogurt
☐ Succo di frutta
☐ Bibite gassate
☐ Thè o tisana
☐ Acqua
☐ Altro ……………………………………

LA CENA

Di solito è costituita da : *(risposte multiple)*

☐ Primo
☐ Secondo
☐ Contorno
☐ Frutta,spremuta o centrifugato di frutta
☐ Dolce
☐ Acqua
☐ Succo di frutta
☐ Bibite gassate
☐ Thè o tisana
☐ Latte/latte aromatizzato (con cacao,…)
☐ Altro ……………………………………

DOPO LA CENA

Suo figlio mangia e beve dopo la cena? ☐ Tutti i giorni (7 volte) ☐ Quasi tutti i giorni (4-6) volte ☐ A volte (1-3 volte) ☐ Mai

Di solito quali **ALIMENTI e BEVANDE** consuma? *(risposte multiple)*

☐ Frutta, spremuta, centrifugato di frutta
☐ Prodotto da forno dolce (biscotti, merendine, brioches, torte,, …)
☐ Prodotto da forno salato (crackers, pizza, crescente, …)
☐ Panino imbottito (con formaggi, salumi, creme spalmabili dolci, salate…)
☐ Snack dolce (cioccolato, barrette, gelati, torrone…)
☐ Snack salato (patatine, pop corn, salatini, soffiati al formaggio…)
☐ Latte/latte aromatizzato (con cacao,…)
☐ Yogurt
☐ Succo di frutta
☐ Bibite gassate
☐ Thè, Tisane, camomilla..
☐ Acqua
☐ Altro (specificare) ……………………………

La preghiamo, ora, di osservare queste immagini e di indicare a quale assomiglia suo/a figlio/a. Figura scelta n°_________

Figlia

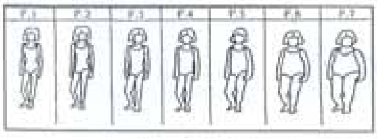

Figlio

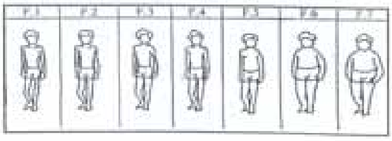

…e a quale figura vorreste che assomigliasse? Figura scelta n°____________

Frequenze di consumo

	Mai	Meno di 1 volta al mese	1-3 volte mese	1 volta a sett.	2-4 volte a sett.	5-6 volte a sett.	1 volta al giorno	2-3 volte al giorno	4-5 volte al giorno
Suo figlio/a mangia **CEREALI**?	☐	☐	☐	☐	☐	☐	☐	☐	☐
Pasta	☐	☐	☐	☐	☐	☐	☐	☐	☐
Riso, orzo	☐	☐	☐	☐	☐	☐	☐	☐	☐
Pane	☐	☐	☐	☐	☐	☐	☐	☐	☐
Grissini/crackers	☐	☐	☐	☐	☐	☐	☐	☐	☐
Pizza/focaccia	☐	☐	☐	☐	☐	☐	☐	☐	☐
Suo figlio/a mangia **CARNE**?	☐	☐	☐	☐	☐	☐	☐	☐	☐
Bovina	☐	☐	☐	☐	☐	☐	☐	☐	☐
Suina	☐	☐	☐	☐	☐	☐	☐	☐	☐
Equina	☐	☐	☐	☐	☐	☐	☐	☐	☐
Pollo, tacchino, coniglio	☐	☐	☐	☐	☐	☐	☐	☐	☐
Altro (struzzo…)	☐	☐	☐	☐	☐	☐	☐	☐	☐
Suo figlio/a mangia **SALUMI** ?	☐	☐	☐	☐	☐	☐	☐	☐	☐
Suo figlio/a mangia **UOVA**?	☐	☐	☐	☐	☐	☐	☐	☐	☐
Suo figlio/a mangia **PESCE**?	☐	☐	☐	☐	☐	☐	☐	☐	☐
Pesci con spina dorsale	☐	☐	☐	☐	☐	☐	☐	☐	☐
Pesce in scatola (tonno, ..)	☐	☐	☐	☐	☐	☐	☐	☐	☐
Molluschi, mitili, crostacei	☐	☐	☐	☐	☐	☐	☐	☐	☐
Bastoncini	☐	☐	☐	☐	☐	☐	☐	☐	☐
Suo figlio/a mangia **LATTE / YOGURT** ?	☐	☐	☐	☐	☐	☐	☐	☐	☐
Suo figlio mangia **FORMAGGI**?	☐	☐	☐	☐	☐	☐	☐	☐	☐
Formaggi freschi	☐	☐	☐	☐	☐	☐	☐	☐	☐
Formaggi stagionati	☐	☐	☐	☐	☐	☐	☐	☐	☐
Suo figlio/a mangia **LEGUMI** (fagioli, lenticchie, piselli, ceci, fave…)	☐	☐	☐	☐	☐	☐	☐	☐	☐
Suo figlio/a mangia **PATATE** ?	☐	☐	☐	☐	☐	☐	☐	☐	☐
Suo figlio/a mangia **VERDURE**?	☐	☐	☐	☐	☐	☐	☐	☐	☐
Verdure in minestrone	☐	☐	☐	☐	☐	☐	☐	☐	☐
Pomodori e/o salsa	☐	☐	☐	☐	☐	☐	☐	☐	☐
Biete/spinaci	☐	☐	☐	☐	☐	☐	☐	☐	☐
Lattuga/insalate	☐	☐	☐	☐	☐	☐	☐	☐	☐
Cavolo/ cavolfiore	☐	☐	☐	☐	☐	☐	☐	☐	☐
Fagiolini	☐	☐	☐	☐	☐	☐	☐	☐	☐
Carote	☐	☐	☐	☐	☐	☐	☐	☐	☐
Peperoni	☐	☐	☐	☐	☐	☐	☐	☐	☐
Carciofi/asparagi	☐	☐	☐	☐	☐	☐	☐	☐	☐
Cipolle/aglio	☐	☐	☐	☐	☐	☐	☐	☐	☐
Finocchi	☐	☐	☐	☐	☐	☐	☐	☐	☐
Zucchine/ melanzane	☐	☐	☐	☐	☐	☐	☐	☐	☐
Suo figlio/a mangia **FRUTTA** ?	☐	☐	☐	☐	☐	☐	☐	☐	☐
Mele, pere	☐	☐	☐	☐	☐	☐	☐	☐	☐
Banane	☐	☐	☐	☐	☐	☐	☐	☐	☐
Agrumi/kiwi	☐	☐	☐	☐	☐	☐	☐	☐	☐
Suo figlio/a mangia **FRUTTA SECCA**?	☐	☐	☐	☐	☐	☐	☐	☐	☐
Suo figlio/a mangia **DOLCI**?	☐	☐	☐	☐	☐	☐	☐	☐	☐
Gelato	☐	☐	☐	☐	☐	☐	☐	☐	☐
Biscotti	☐	☐	☐	☐	☐	☐	☐	☐	☐
Merendine	☐	☐	☐	☐	☐	☐	☐	☐	☐
Torte/paste	☐	☐	☐	☐	☐	☐	☐	☐	☐
Creme spalmabili (tipo "Nutella",..)	☐	☐	☐	☐	☐	☐	☐	☐	☐
Cioccolato, barrette									
Miele/marmellata	☐	☐	☐	☐	☐	☐	☐	☐	☐
Suo figlio/a mangia **DOLCIUMI**? (caramelle, gomme, ..)	☐	☐	☐	☐	☐	☐	☐	☐	☐
Suo figlio mangia **SNACK SALATI** (patatine, popcorn, salatini)?	☐	☐	☐	☐	☐	☐	☐	☐	☐
Suo figlio/a **BEVE**?	☐	☐	☐	☐	☐	☐	☐	☐	☐
Acqua	☐	☐	☐	☐	☐	☐	☐	☐	☐
Bibite gassate	☐	☐	☐	☐	☐	☐	☐	☐	☐
Spremute/succhi	☐	☐	☐	☐	☐	☐	☐	☐	☐
Tisane, thè	☐	☐	☐	☐	☐	☐	☐	☐	☐
Bevande energizzanti	☐	☐	☐	☐	☐	☐	☐	☐	☐

Dieta & Salute

Per ciascuna domanda la preghiamo di leggere sempre fino <u>in fondo</u> tutte le risposte.

A) Quali sono, nella Vostra famiglia, le principali fonti di informazione in tema alimentare ? (Massimo 5 risposte)

1 ☐ Servizio Sanitario Nazionale (Pediatra, Medico di Base, dietista,..)
2 ☐ Scuola
3 ☐ Pubblica Amministrazione(Regione, Provincia, Comune)
4 ☐ Associazioni dei consumatori-
5 ☐ Libri, testi specializzati
6 ☐ Articoli su giornali (quotidiani, settimanali, mensili…)
7 ☐ Negozi specializzati (farmacia, erboristeria,…)
8 ☐ Pubblicità
9 ☐Confezione/etichetta
10 ☐ Colleghi
11 ☐ Palestre/centri di bellezza/ dimagrimento
12 ☐ Grande distribuzione (supermarket..)
13 ☐ Materiale informativo industrie alimentari
14 ☐ TV/radio
15 ☐ Internet
16 ☐ Altro (Specificare) ……………………………………
……………………………………………………

B) Quali fattori influenzano le scelte alimentari nella Vostra famiglia? (Massimo 5 risposte)

1 ☐ Sapere "cosa c'è dentro"
2 ☐ Abitudine o routine
3 ☐ Prezzo
4 ☐ Quello che tutta la famiglia vuole mangiare
5 ☐ Radici culturali/religiose/etniche
6 ☐ Gusto/sapore dei cibi
7 ☐ Tentare di mangiar sano/equilibrato
8 ☐ Facilità di preparazione
9 ☐ Dimagrire
10 ☐ Presentazione, confezione, involucro
11 ☐ Prodotto dietetico
12 ☐ Moda, pubblicità
13 ☐ Dieta prescritta (medico,dietologo,allenatore)
14 ☐ Contenuto di additivi, conservanti coloranti
15 ☐ Sicurezza alimentare
16 ☐ Essere vegetariano o altra cultura alimentare
17 ☐ Prodotti biologici, lotta integrata
18 ☐ Altro (specificare)……………………………………

C) Quali cambiamenti alimentari la Vostra famiglia ha apportato dalla nascita del bambino? (Massimo 5 risposte)

1 ☐ Minor consumo di zuccheri/cibi dolci
2 ☐ Eliminare cibi con additivi, conservanti, coloranti
3 ☐ Minor consumo di grassi/cibi grassi
4 ☐ Ridurre le quantità
5 ☐ Maggior consumo di frutta e verdura
6 ☐ Bere più acqua
7 ☐ Mangiare una più ampia varietà di cibi di prodotti light/dietetici
8 ☐ Minor consumo di carne
9 ☐ Maggior consumo di carne
11 ☐ Minor consumo di pane, pasta
12 ☐ Minor consumo di tea/caffè
13 ☐ Maggior consumo di formaggi, latte, yogurt
14 ☐ Minor consumo di formaggi, latte, yogurt
15 ☐ Evitare di mangiare tra i pasti
16 ☐ Minor consumo di sale
17 ☐ Maggior consumo di cibi ricchi di fibre
18 ☐ Maggior consumo di pesce
19 ☐ Altro(specificare)……………………………………
……………………………………………………

D)Quali sono , nella Vostra famiglia, maggiori difficoltà/barriere al "mangiar più sano"? (Massimo 5 risposte)

1 ☐ Orari
2 ☐ I cibi sani sono poco desiderabili
3 ☐ Vita molto impegnata, poco tempo per preparare/consumare i cibi
4 ☐ Preferenze gusti della famiglia
5 ☐ Un cambiamento troppo grande rispetto al nostro stile alimentare
6 ☐ Prezzo dei cibi sani
7 ☐ I cibi sani sono più deperibili
8 ☐ Gli esperti cambiano spesso parere
9 ☐ Scarsa conoscenza sul mangiar sano
10 ☐ Scarse abilità culinarie
11 ☐ Maggior tempo nella preparazione dei cibi sani
12 ☐ Non voler cambiare
13 ☐ Mancanza di forza di volontà
14 ☐ Altro(specificare)……………………………………
……………………………………………………
……………………………………………………

In Cucina e a Tavola

Chi prepara in genere i pasti casalinghi? (risposte multiple)
☐ Madre ☐ Padre ☐Fratelli/sorelle
☐Nonno ☐ Nonna ☐Chi capita
☐ Altro (specificare)……………………………………

Tutta la famiglia si riunisce a tavola nei giorni feriali:
(risposte multiple) ☐ A colazione ☐ A pranzo ☐ A cena

Quante volte "*si mangia fuori*" con la famiglia: (una risposta)
☐Almeno una volta alla settimana ☐Una o due volte al mese
☐Solo in particolari ricorrenze ☐Sporadicamente ☐Mai

La televisione è accesa durante i pasti:
☐Mai ☐Talvolta ☐Spesso

Quali sono gli ostacoli per un maggior consumo di:

(risposte multiple)	legumi	frutta	verdura	pesce
Costo elevato	☐	☐	☐	☐
Non piacciono	☐	☐	☐	☐
Occorre troppo tempo di preparazione	☐	☐	☐	☐
Non sono sempre disponibili in casa	☐	☐	☐	☐
In famiglia nessuno li mangia	☐	☐	☐	☐

Per la preparazione dei pasti in genere vengono utilizzati: (risposte multi[illegible]
☐Prodotti freschi ☐Alimenti biologici, a lotta integrata…..
☐Piatti già pronti ☐Alimenti speciali (integrali, a base di soia…)
☐Alimenti surgelati ☐Alimenti arricchiti (con omega 3….)
☐Alimenti in busta ☐Alimenti inscatolati
☐Altro (specificare)……………………………………

Che tipo di grasso si usa in famiglia per la <u>cottura</u> dei cibi, cioè a caldo?
☐ Abitualmente grassi di origine animale (burro, pancetta, panna…)
☐ Alterno grassi animali e oli vegetali
☐ Quasi esclusivamente oli vegetali (semi, oliva)
☐ Solo olio di oliva

Che tipo di grasso si usa in famiglia per <u>condire</u> i cibi, cioè a crudo?
☐ Abitualmente grassi di origine animale (burro, pancetta, panna…)
☐ Alterno grassi animali e oli vegetali
☐ Quasi esclusivamente oli vegetali (semi, oliva)
☐ Solo olio di oliva

Fa uso di sale iodato? ☐ Si, ☐ No
Se NO, ne ha sentito parlare ? ☐ Si, ☐ No

Propone a suo figlio piatti dei paesi d'origine della vostra famiglia? ☐ Mai ☐Qualche volta ☐ Spesso
Quali?(specificare)……………………………………
……………………………………………………

Propone a suo figlio piatti della tradizione/cucina locale? ☐ Mai ☐ Qualche volta ☐ Spesso
Quali?(specificare)……………………………………
……………………………………………………

Dieta & Salute

Per ciascuna domanda la preghiamo di leggere sempre fino in fondo tutte le risposte.

A) Quali sono, nella Vostra famiglia, le principali fonti di informazione in tema alimentare ? (Massimo 5 risposte)

1 ☐ Servizio Sanitario Nazionale (Pediatra, Medico di Base, dietista,..)
2 ☐ Scuola
3 ☐ Pubblica Amministrazione(Regione, Provincia, Comune)
4 ☐ Associazioni dei consumatori-
5 ☐ Libri, testi specializzati
6 ☐ Articoli su giornali (quotidiani, settimanali, mensili…)
7 ☐ Negozi specializzati (farmacia, erboristeria,…)
8 ☐ Pubblicità
9 ☐Confezione/etichetta
10 ☐ Colleghi
11 ☐ Palestre/centri di bellezza/ dimagrimento
12 ☐ Grande distribuzione (supermarket..)
13 ☐ Materiale informativo industrie alimentari
14 ☐ TV/radio
15 ☐ Internet
16 ☐ Altro (Specificare) ……………………………………
…………………………………………………………………

B) Quali fattori influenzano le scelte alimentari nella Vostra famiglia? (Massimo 5 risposte)

1 ☐ Sapere "cosa c'è dentro"
2 ☐ Abitudine o routine
3 ☐ Prezzo
4 ☐ Quello che tutta la famiglia vuole mangiare
5 ☐ Radici culturali/religiose/etniche
6 ☐ Gusto/sapore dei cibi
7 ☐ Tentare di mangiar sano/equilibrato
8 ☐ Facilità di preparazione
9 ☐ Dimagrire
10 ☐ Presentazione, confezione, involucro
11 ☐ Prodotto dietetico
12 ☐ Moda, pubblicità
13 ☐ Dieta prescritta (medico,dietologo,allenatore)
14 ☐ Contenuto di additivi, conservanti coloranti
15 ☐ Sicurezza alimentare
16 ☐ Essere vegetariano o altra cultura alimentare
17 ☐ Prodotti biologici, lotta integrata
18 ☐ Altro (specificare)……………………………………

C) Quali cambiamenti alimentari la Vostra famiglia ha apportato dalla nascita del bambino? (Massimo 5 risposte)

1 ☐ Minor consumo di zuccheri/cibi dolci
2 ☐ Eliminare cibi con additivi, conservanti, coloranti
3 ☐ Minor consumo di grassi/cibi grassi
4 ☐ Ridurre le quantità
5 ☐ Maggior consumo di frutta e verdura
6 ☐ Bere più acqua
7 ☐ Mangiare una più ampia varietà di cibi di prodotti light/dietetici
8 ☐ Minor consumo di carne
9 ☐ Maggior consumo di carne
10 ☐ Evitare di saltare i pasti
11 ☐ Minor consumo di pane, pasta
12 ☐ Minor consumo di tea/caffè
13 ☐ Maggior consumo di formaggi, latte, yogurt
14 ☐ Minor consumo di formaggi, latte, yogurt
15 ☐ Evitare di mangiare tra i pasti
16 ☐ Minor consumo di sale
17 ☐ Maggior consumo di cibi ricchi di fibre
18 ☐ Maggior consumo di pesce
19 ☐ Altro(specificare)……………………………………
…………………………………………………………………

D)Quali sono , nella Vostra famiglia, maggiori difficoltà/barriere al "mangiar più sano"? (Massimo 5 risposte)

1 ☐ Orari
2 ☐ I cibi sani sono poco desiderabili
3 ☐ Vita molto impegnata, poco tempo per preparare/consumare i cibi
4 ☐ Preferenze gusti della famiglia
5 ☐ Un cambiamento troppo grande rispetto al nostro stile alimentare
6 ☐ Prezzo dei cibi sani
7 ☐ I cibi sani sono più deperibili
8 ☐ Gli esperti cambiano spesso parere
9 ☐ Scarsa conoscenza sul mangiar sano
10 ☐ Scarse abilità culinarie
11 ☐ Maggior tempo nella preparazione dei cibi sani
12 ☐ Non voler cambiare
13 ☐ Mancanza di forza di volontà
14 ☐ Altro(specificare)……………………………………
…………………………………………………………………
…………………………………………………………………

In Cucina e a Tavola

Chi prepara in genere i pasti casalinghi? (risposte multiple)
☐ Madre ☐ Padre ☐Fratelli/sorelle
☐Nonno ☐ Nonna ☐Chi capita
☐ Altro (specificare)……………………………………

Tutta la famiglia si riunisce a tavola nei giorni feriali:
(risposte multiple) ☐ A colazione ☐ A pranzo ☐ A cena

Quante volte "*si mangia fuori*" con la famiglia: (una risposta)
☐Almeno una volta alla settimana ☐Una o due volte al mese
☐Solo in particolari ricorrenze ☐Sporadicamente ☐Mai

La televisione è accesa durante i pasti:
☐Mai ☐Talvolta ☐Spesso

Quali sono gli ostacoli per un maggior consumo di:
(risposte multiple)

	legumi	frutta	verdura	pesce
Costo elevato	☐	☐	☐	☐
Non piacciono	☐	☐	☐	☐
Occorre troppo tempo di preparazione	☐	☐	☐	☐
Non sono sempre disponibili in casa	☐	☐	☐	☐
In famiglia nessuno li mangia	☐	☐	☐	☐

Per la preparazione dei pasti in genere vengono utilizzati: (risposte multiple)
☐Prodotti freschi ☐Alimenti biologici, a lotta integrata…..
☐Piatti già pronti ☐Alimenti speciali (integrali, a base di soia…)
☐Alimenti surgelati ☐Alimenti arricchiti (con omega 3….)
☐Alimenti in busta ☐Alimenti inscatolati
☐Altro (specificare)……………………………………

Che tipo di grasso si usa in famiglia per la cottura dei cibi, cioè a caldo?
☐ Abitualmente grassi di origine animale (burro, pancetta, panna…)
☐ Alterno grassi animali e oli vegetali
☐ Quasi esclusivamente oli vegetali (semi, oliva)
☐ Solo olio di oliva

Che tipo di grasso si usa in famiglia per condire i cibi, cioè a crudo?
☐ Abitualmente grassi di origine animale (burro, pancetta, panna…)
☐ Alterno grassi animali e oli vegetali
☐ Quasi esclusivamente oli vegetali (semi, oliva)
☐ Solo olio di oliva

Fa uso di sale iodato? ☐ Si, ☐ No
Se NO, ne ha sentito parlare ☐ Si, ☐ No

Propone a suo figlio piatti della tradizione locale? ☐ Mai ☐Qualche volta ☐ Spesso
Quali?(specificare massimo due)……………………………………
Propone a suo figlio piatti dei paesi d'origine della vostra famiglia? ☐ Mai ☐ Qualche volta ☐ Spesso
Quali?(specificare massimo due)……………………………………

Allegato n°3
Questionario somministrato ai bambini di 9 anni

Allegato n°3
Questionario somministrato ai bambini di 9 anni

SERVIZIO SANITARIO REGIONALE EMILIA-ROMAGNA — Regione Emilia Romagna ☐☐☐☐☐☐☐☐

Sorveglianza Nutrizionale Infanzia Adolescenza	Scuola____________ Comune____________<br>Data di compilazione: ____/____/____<br>Sesso: ☐Maschio ☐Femmina Data di nascita: ___/___/___<br>Peso__________Kg Altezza__________cm

Quanto pensi di sapere sulla nutrizione? *(Una sola risposta)*
☐So molto ☐ So alcune cose ☐ So poco ☐ Non so nulla

Come ti piacerebbe imparare la nutrizione? *(Puoi dare fino a 3 risposte)*
☐ Giornalini/Riviste
☐ Laboratorio di cucina
☐ Laboratorio di educazione al gusto
☐ Programmi TV
☐ Materia scolastica
☐ Materiale informativo (filmati,CD,,opuscoli...)
☐ Visite guidate in fattoria
Altro*(specificare)*__________

Penso che dovrei mangiare	di più	di meno	uguale
Bevande zuccherate(aranciate, cola, te' confezionato ec)	☐	☐	☐
Carne	☐	☐	☐
Cioccolato / crema di cioccolato	☐	☐	☐
Dolci da forno (biscotti, merendine, torte,...)	☐	☐	☐
Dolciumi (caramelle, gomme,....)	☐	☐	☐
Formaggi	☐	☐	☐
Frullati / Spremute	☐	☐	☐
Frutta	☐	☐	☐
Gelato	☐	☐	☐
Latte e yogurt	☐	☐	☐
Legumi (piselli, fagioli,lenticchie,ceci...)	☐	☐	☐
Minestre di verdure	☐	☐	☐
Pane	☐	☐	☐
Pasta / Riso	☐	☐	☐
Patate	☐	☐	☐
Patatine (e simili) confezionate/fast food	☐	☐	☐
Pesce	☐	☐	☐
Pomodori	☐	☐	☐
Succhi di frutta	☐	☐	☐
Uova	☐	☐	☐
Verdure cotte	☐	☐	☐
Verdure crude	☐	☐	☐

Quali pasti sei abituato a consumare ogni giorno ?
☐ Colazione ☐ Merenda ☐ Pranzo ☐ Merenda ☐ Cena ☐ Dopo cena ☐ Fuori pasto

A tuo parere quali pasti dovresti consumare nella giornata per crescere sano ?
☐ Colazione ☐ Merenda ☐ Pranzo ☐ Merenda ☐ Cena ☐ Dopo cena ☐ Fuori pasto

Puoi dirmi se sei d'accordo o no con le affermazioni seguenti?	SI	NO	Non sc
E' importante mangiare cibi come pane e cereali integrali	☐	☐	☐
Per rimanere sano devi mangiare meno grassi	☐	☐	☐
Il latte fa le ossa forti	☐	☐	☐
Va bene mangiare cibi come caramelle e gelato, ma non tutti i giorni	☐	☐	☐
Andare tutte le settimane al fast-food va bene	☐	☐	☐
E' meglio mangiare piccole quantità di cibi differenti anziché molto di un cibo sol	☐	☐	☐
La tua salute durante la crescita dipende dal cibo che mangi	☐	☐	☐
Frutta e verdura conservata sono nutrienti quanto frutta e verdura fresca	☐	☐	☐
Va bene mangiare il cioccolato ogni giorno	☐	☐	☐
Le bibite gassate dissetano come l'acqua.	☐	☐	☐
Per crescere sano lo sport e l'attività motoria sono importanti come il cibo chee mangi	☐	☐	☐

Per rimanere sano il tuo corpo ha bisogno di.....(E possibile barrare più di una risposta)

☐Proteine ☐Vitamine ☐Calorie ☐Amidi
☐Grassi ☐Sale da cucina ☐Calcio ☐Preparazioni a base di Vitamine/minerali
☐Ferro ☐Fibre ☐Acqua ☐Zucchero aggiunto

Ti fai guidare nell'alimentazione da... (Puoi dare fino a 3 risposte)

☐ la pubblicità ☐ i genitori , la famiglia
☐ l'abitudine ☐ i compagni, gli amici
☐ il sapore ☐ non so
☐ "sorpresine" , raccolta punti.... altro..............................

Rifletti e poi rispondi alle affermazioni seguenti:	Vero	Falso
Mangio soprattutto cibi ricchi e appetitosi	☐	☐
E' molto difficile farmi variare l'alimentazione	☐	☐
Mi piace fare tanti piccoli spuntini nell'arco della giornata	☐	☐
Mi piace un'alimentazione molto varia	☐	☐
Farmi mangiare è sempre un'impresa molto difficile	☐	☐
Bado più alla quantità che alla qualità del cibo	☐	☐
Amo mangiare sempre le stesse cose	☐	☐
Mangio soprattutto fuori pasto	☐	☐
Mangio volentieri tutto ciò che mi danno	☐	☐
E' necessario usare molta fantasia per stimolarmi a mangiare	☐	☐

Secondo te si hanno benefici da un'alimentazione ricca di frutta e verdura? ☐ SI ☐ NO
Se SI, indicare quali (Puoi dare fino a 3 risposte)

☐ Non fanno ingrassare ☐ Sono ricchi di fibra
☐ Fanno dimagrire ☐ Sono ricchi di vitamine e sali minerali
☐ Aiutano a star bene Altro (indicare cosa) ....

Sei sano quando...(Puoi dare fino a 3 risposte)

☐non ti ammali ☐giochi volentieri ☐dormi bene ☐sei sereno ☐hai appetito ☐ sei attivo

Per favore, scegli una risposta per ogni cibo o bevanda	Fa molto bene alla salute	Fa bene alla salute	Non fa bene alla salute	TI PIACE	Mai assaggiato	Molto	Così così	Per niente
Acqua	☐	☐	☐	Legumi (piselli, fagioli, lenticchie)	?	☺	😐	☹
Bibite zuccherate (aranciata cola, te' confezionato, ecc)	☐	☐	☐	Broccoli	?	☺	😐	☹
Biscotti	☐	☐	☐	Carciofi	?	☺	😐	☹
Burro	☐	☐	☐	Carote	?	☺	😐	☹
Dolciumi (caramelle, gomme	☐	☐	☐	Cavolfiore	?	☺	😐	☹
Carne	☐	☐	☐	Pomodoro	?	☺	😐	☹
Cereali per colazione	☐	☐	☐	Fagiolini	?	☺	😐	☹
Cioccolato-crema di cioccol	☐	☐	☐	Finocchi	?	☺	😐	☹
Formaggio	☐	☐	☐	Funghi	?	☺	😐	☹
Frutta	☐	☐	☐	Insalata	?	☺	😐	☹
Gelato	☐	☐	☐	Peperone	?	☺	😐	☹
Hamburgers (fast food)	☐	☐	☐	Patata	?	☺	😐	☹
Latte / yogurt	☐	☐	☐	Sedano	?	☺	😐	☹
Legumi(fagioli, ceci, piselli...)	☐	☐	☐	Spinaci	?	☺	😐	☹
Merendine	☐	☐	☐	Zucchine	?	☺	😐	☹
Minestrone	☐	☐	☐	Agrumi	?	☺	😐	☹
Olio d'oliva	☐	☐	☐	Banane	?	☺	😐	☹
Pane	☐	☐	☐	Kiwi	?	☺	😐	☹
Pasta, riso	☐	☐	☐	Frutti di Bosco	?	☺	😐	☹
Patate	☐	☐	☐	Frutta Esotica	?	☺	😐	☹
Patatine confezionate	☐	☐	☐	Frutta Secca Guscio	?	☺	😐	☹
Pesce	☐	☐	☐	Pera Mela	?	☺	😐	☹
Pizza	☐	☐	☐	Melone	?	☺	😐	☹
Prosciutto cotto	☐	☐	☐	Uva	?	☺	😐	☹
Prosciutto crudo	☐	☐	☐	Albicocche Pesche	?	☺	😐	☹
Salame/Mortadella	☐	☐	☐	Bastoncini	?	☺	😐	☹
Succo di frutta	☐	☐	☐	Gamberi/Gamberett	?	☺	😐	☹
Torte	☐	☐	☐	Sogliola/ Platessa	?	☺	😐	☹
Uova	☐	☐	☐	Vongole, Cozze	?	☺	😐	☹
Verdura cruda	☐	☐	☐	Pesce: trancio/filet	?	☺	😐	☹
Verdura cotta	☐	☐	☐	Polpo Seppia	?	☺	😐	☹
Vino				Tonno In Scatola	?	☺	😐	☹

Quante volte al giorno ti lavi i denti? *(Una sola risposta)*

☐ meno di una volta al giorno ☐ dopo i pasti principali (colazione, pranzo e cena)

☐ una volta al giorno ☐ due volte al giorno

Per quanto tempo ti spazzoli i denti ? *(Una sola risposta)*

☐ non so ☐ meno di un minuto ☐ più di un minuto ☐ più di due minuti

Cosa usi per pulire i denti oltre allo spazzolino? *(puoi dare più risposte)*

☐ il filo interdentale ☐ dentifricio ☐ altro ....

Rifletti e poi rispondi alle affermazioni seguenti:

	Non fa venire la carie	Fa venire la carie		Non fa venire la carie	Fa venire la carie
Acqua	☐	☐	Legumi	☐	☐
Barrette/Cioccolata	☐	☐	Miele/marmellata	☐	☐
Bevande dolci (te, succhi, cola,......)	☐	☐	"Nutella", creme spalmabili	☐	☐
Biscotti e Merendine	☐	☐	Olii/Burro	☐	☐
Caramelle/gomme da masticare	☐	☐	Pane	☐	☐
Caramelle/gomme da masticare senza zucchero	☐	☐	Paste	☐	☐
Carne	☐	☐	Pesce	☐	☐
Formaggi	☐	☐	Torte	☐	☐
Frutta	☐	☐	Uova	☐	☐
Gelato	☐	☐	Verdura	☐	☐
Latte	☐	☐	Zucchero (aggiunto)	☐	☐

Quale di queste figure è più simile a te ora? Figura scelta n° ....

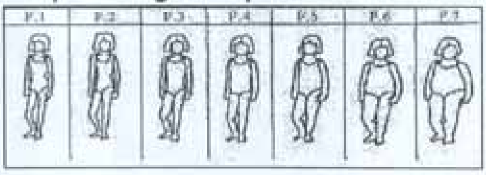

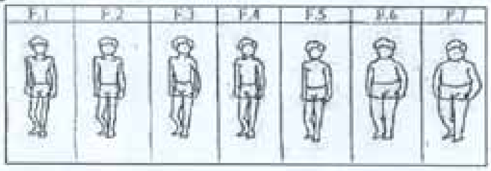

A quale di queste figure vorresti assomigliare? Figura scelta........

Quale di queste figure è più simile al tuo papà e alla tua mamma ora ?

PAPA' Figura scelta:.... **Mamma Figura scelta** ....

F1 F2 F3 F4 F5

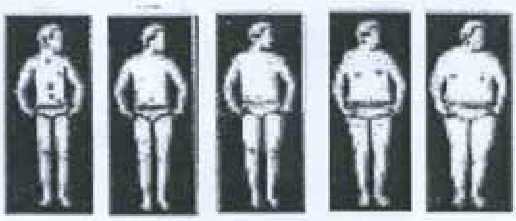

F1 F2 F3 F4 F5

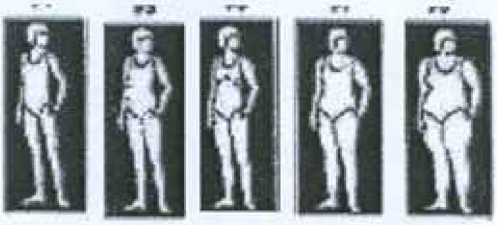

A quale di queste figure vorresti che assomigliasse Figura scelta.......

PAPA' Figura scelta:.... **Mamma Figura scelta** ....

AZIENDA U.S.L. di CESENA
Centro Stampa
